# Targeting Peripherally Restricted Cannabinoid Receptor 1, Cannabinoid Receptor 2, and Endocannabinoid-Degrading Enzymes for the Treatment of Neuropathic Pain Including Neuropathic Orofacial Pain

**DOI:** 10.3390/ijms21041423

**Published:** 2020-02-20

**Authors:** Mohammad Zakir Hossain, Hiroshi Ando, Shumpei Unno, Junichi Kitagawa

**Affiliations:** 1 Department of Oral Physiology, School of Dentistry, Matsumoto Dental University, 1780 Gobara Hirooka, Shiojiri, Nagano 399-0781, Japan; shumpei.unno@mdu.ac.jp (S.U.); junichi.kitagawa@mdu.ac.jp (J.K.); 2 Department of Biology, School of Dentistry, Matsumoto Dental University, 1780 Gobara, Hirooka, Shiojiri, Nagano 399-0781, Japan; hiroshi.ando@mdu.ac.jp

**Keywords:** neuropathic pain, neuropathic orofacial pain, CB2-selective agonists, peripherally restricted CB1 agonists, endocannabinoid-degrading enzyme inhibitors

## Abstract

Neuropathic pain conditions including neuropathic orofacial pain (NOP) are difficult to treat. Contemporary therapeutic agents for neuropathic pain are often ineffective in relieving pain and are associated with various adverse effects. Finding new options for treating neuropathic pain is a major priority in pain-related research. Cannabinoid-based therapeutic strategies have emerged as promising new options. Cannabinoids mainly act on cannabinoid 1 (CB1) and 2 (CB2) receptors, and the former is widely distributed in the brain. The therapeutic significance of cannabinoids is masked by their adverse effects including sedation, motor impairment, addiction and cognitive impairment, which are thought to be mediated by CB1 receptors in the brain. Alternative approaches have been developed to overcome this problem by selectively targeting CB2 receptors, peripherally restricted CB1 receptors and endocannabinoids that may be locally synthesized on demand at sites where their actions are pertinent. Many preclinical studies have reported that these strategies are effective for treating neuropathic pain and produce no or minimal side effects. Recently, we observed that inhibition of degradation of a major endocannabinoid, 2-arachydonoylglycerol, can attenuate NOP following trigeminal nerve injury in mice. This review will discuss the above-mentioned alternative approaches that show potential for treating neuropathic pain including NOP.

## 1. Introduction

Neuropathic pain is defined as pain caused by a lesion or disease of the somatosensory nervous system [1,2]. It is a health problem that has negative impacts on both the individual quality of life and the community [3]. It is associated with significant societal costs resulting from greater healthcare utilization, disability, and loss of productivity [4,5]. Neuropathic pain in the orofacial regions (e.g., head, neck, face, oral, or perioral regions) can be termed as neuropathic orofacial pain (NOP) and may arise from nerve compression or injury to peripheral nerves during dental operative procedures, such as tooth extraction, root canal treatment, and dental implant surgery (e.g., trigeminal neuralgia, post-traumatic trigeminal neuropathy) as well as from systemic diseases (e.g., diabetic neuropathy), viral infections (e.g., trigeminal post-herpetic neuralgia) and neurovascular diseases (e.g., tension type headache, chronic/episodic migraine) [6,7,8,9,10,11,12]. NOP may be characterized by spontaneous pain (ongoing or episodic), pain resulting from stimuli that would not normally provoke pain (allodynia) and exaggerated pain responses to noxious stimuli (hyperalgesia) [6,7,8,9]. Patients with poorly controlled neuropathic pain have significantly poorer health status and increased symptoms of anxiety and depression [4,5]. The mechanisms of neuropathic pain are complex, rendering it a challenge for clinicians to treat effectively [13,14,15]. Tricyclic antidepressants (e.g., nortriptyline, desipramine), serotonin–noradrenaline reuptake inhibitors (e.g., duloxetine), and anticonvulsants (e.g., gabapentin, pregabalin) are currently used as first-line treatments for neuropathic pain; however, many patients report incomplete relief of pain as well as adverse effects of these drugs such as cardiotoxicity, dry mouth, orthostatic hypotension, constipation, and dizziness [13,14]. Topical lidocaine (which only acts locally) and opioids (e.g., morphine) are considered as second- and third-line drugs for treatment of neuropathic pain. Opioids have adverse effects including sedation, constipation, physical dependence, respiratory depression tolerance, and addiction [13,14,16], and they have a high degree of abuse potential [17]. The management of neuropathic pain with these contemporary pharmacotherapies exhibits a high failure rate [13,14,15]. These treatment failures may occur because of lack of analgesic efficacy, intolerance, contraindications to various classes of medications and the presence of side effects [4,5,13,14,15]. Therefore, a major priority in pain-related research is to identify new therapeutic strategies for treating neuropathic pain. In recent decades, cannabinoid- and endocannabinoid-based therapeutic strategies for neuropathic pain treatment have gained popularity [18,19,20,21,22,23].

Cannabinoids are chemical compounds found in *Cannabis* plants (*Cannabis sativa*, *Cannabis indica,* and *Cannabis ruderalis)*. These plants contain many natural compounds. Among them, more than 100 compounds are currently considered cannabinoids [24], although the full characterization of all of these compounds is still lacking. Delta-9-tetrahydrocannabinol (THC) and cannabidiol (CBD) are the first two cannabinoids that were chemically characterized from flowers of *Cannabis sativa* [25,26]. THC is a psychoactive substance, whereas CBD is non-psychoactive [27]. The evidence for use of *Cannabis sativa* for medicinal purposes dates to before the Christian era in Asia. It was used as an analgesic for toothache, headache, and neuralgia as early as 1000 B.C. in Indian Ayurvedic medicine [28].

Endocannabinoids are cannabis-like molecules synthesized in the body [29]. The two primary endocannabinoids in the body are 2-arachydonoylglycerol (2-AG) and N-arachidonoyl ethanolamine (AEA) [29]. AEA appears to be produced from N-acyl-phosphatidylethanolamine (NAPE) by NAPE-specific phospholipase D (NAPE-PLD) or via other routes not involving NAPE-PLD, and 2-AG is produced from diacylglycerol by the action of diacylglycerol lipase [30,31,32,33,34,35]. Endocannabinoids are synthesized and released on demand locally in response to physiological and pathological stimuli [21,30,31,32,33,34,35]. They are degraded by hydrolyzing enzymes. AEA is mainly degraded by fatty acid amide hydrolase (FAAH), and 2-AG is mainly degraded by monoacylglycerol lipase (MAGL) [30,31,32,33,34,35], although other enzymes may also be involved in 2-AG and AEA degradation [30,31,32,33,34,35].

Both cannabinoids and endocannabinoids mainly act on widely distributed cannabinoid 1 (CB1) and cannabinoid 2 (CB2) receptors, which are G protein-coupled receptors (GPCRs) [36,37]. They may also act on other non-CB1 and non-CB2 cannabinoid-related GPCRs (e.g., GPR18 and GPR55) [38]. Along with cannabinoid receptors, AEA has been observed to act on other receptors implicated in pain processing such as transient receptor potential vanilloid 1 (TRPV1) receptors and peroxisome proliferator-activated receptors (PPARs) [39,40]. The CB1 receptor was discovered in 1990 [37], and the CB2 receptor was discovered in 1993 [36]. Activation of CB1 receptors inhibits adenylyl cyclase, blocks voltage-gated Ca^2+^ channels (VGCCs) and activates K^+^-channels in mammalian neurons, whereas activation of CB2 receptors inhibits adenylyl cyclase but does not block VGCCs or activate K^+^-channels [41,42,43,44,45]. CB1 receptors are predominantly expressed in a wide area of the brain [46], while CB2 receptors are predominantly expressed in immune cells [36,47]. Studies have shown that cannabinoid receptors are distributed in many important sites of pain pathways in the central (CNS) and peripheral nervous system (PNS) including the peripheral and central terminals of primary afferents, peripheral ganglia such as the dorsal root ganglia (DRG), the trigeminal ganglia (TG), second-order neurons in the spinal cord/brainstem, pain-regulatory circuits in the brainstem (e.g., periaqueductal gray, PAG) and different brain regions [48,49,50,51,52].

Various studies have reported that natural and synthetic cannabinoids are effective in the attenuation of acute and chronic pain including neuropathic pain [23,53,54,55,56]. However, the major drawback of using cannabinoids for pain relief is their side effects (cannabimimetic side effects), including sedation, catalepsy (the body becomes stiff), hypothermia, addiction, hypo-locomotion or motor impairment, cognitive impairment and psychological problems [57,58,59]. These cannabimimetic side effects are thought to arise mainly because of global activation of the widespread distribution of CB1 receptors in the brain [57,58,59,60].

To overcome this problem, several alternative strategies have been developed. One strategy is to target CB1 receptors localized in the peripheral tissues [61,62,63,64]. CB1 agonists with limited or no ability to pass the blood–brain barrier have been developed for this purpose and tested in preclinical animal models [63,64]. Another strategy is to selectively target CB2 receptors because they are predominantly expressed outside of the brain. Studies have reported that CB2 receptor agonists attenuated inflammatory and neuropathic pain [65,66]. Another promising alternative strategy for achieving analgesia is to target endocannabinoids [19,20,67,68,69]. In certain disorders, including neuropathic and inflammatory pain, convincing evidence exists regarding increases in endocannabinoids in certain body regions [19,20,21,67,68]. Exaggerated neuronal activity developed under neuropathic pain conditions may increase the synthesis of endocannabinoids at certain locations of the pain pathway [21,70]. This increase in endocannabinoids may be caused by the body’s autoprotective/defense mechanism; however, the rapid cellular uptake and subsequent degradation of endocannabinoids tends to limit the level of analgesia achieved by endocannabinoids [21,61,68,69]. Reducing the degradation of endocannabinoids by inhibiting their degrading enzymes can elevate their levels at sites where their actions are pertinent and produce analgesia [21,61,68,69]. This strategy of increasing endocannabinoids has the benefit of activation of cannabinoid receptors at sites of pain pathways with high endocannabinoid turnover, rather than global activation of CB1 receptors, which can result in side effects [21,61,68,69].

Analgesic effects of various natural and synthetic cannabinoids for neuropathic pain have widely described and reviewed [18,20,23,53,54]. The presence of cannabimimetic side effects is one of the drawbacks of non-selective cannabinoids due to the widespread distribution of the CB1 receptors in the brain [19,20]. Alternative strategies have developed to overcome this problem by using peripherally acting CB1 receptor agonists, selective CB2 receptor agonists, and endocannabinoid degrading enzyme inhibitors [19,20,21]. These strategies are also applicable to NOP. In this review, we will discuss the potential of these alternative strategies for the treatment of neuropathic pain including NOP.

## 2. Targeting Peripherally Restricted CB1 Receptors for the Treatment of Neuropathic Pain

CB1 may be the most abundant and widespread receptor in the mammalian brain [46] and thus may be responsible for the psychological side effects of cannabinoids, which can penetrate the blood–brain barrier. These receptors are observed in a wide area of the brain including the regions involved in pain transmission and modulation, such as the PAG, rostral ventral medulla (RVM), thalamus and amygdala [46,71,72]. They are also found in regions of the spinal cord and brainstem that are involved in pain processing including the spinal dorsal horn, spinal trigeminal nucleus caudalis (Vc) and nerve fibers of the spinal trigeminal tract [46,71,72,73,74,75]. In the PNS, CB1 receptor expression is found in the peripheral nerves including the cell bodies of the primary afferent neurons located in the peripheral ganglia (e.g., DRG, TG) [48,49,50,51,52]. These receptors are localized in both nociceptive and non-nociceptive primary afferent neurons [48,49,50,51,52]. In the TG, CB1 receptors are observed in medium and large diameter neurons of the maxillary and mandibular branches of the trigeminal nerve [50]. In the DRG, they are observed in small, medium and large sized neurons [48,49,51,73,76]. One study reported that CB1 receptors are expressed in 76%–83% of nociceptive neurons in the DRG [77]. They were co-expressed with nociceptive markers such as isolectin B4, TRPV1 and calcitonin gene related peptide [48,51,74,77]. Selective deletion of CB1 receptors from nociceptive neurons of the PNS but not from the CNS substantially reduced the analgesic effect of local and systemic, but not intrathecal, delivery of cannabinoids, which confirmed the contribution of CB1 receptors to nociceptors involved in the analgesic effects of cannabinoids [52]. CB1 receptors are also expressed in both unmyelinated and myelinated nerve fibers of the skin [78].

### 2.1. Modulation of CB1 Receptor Expression under Neuropathic Pain Conditions

Many studies have reported that under inflammatory and neuropathic pain conditions, CB1 receptors are upregulated at various sites of the pain processing pathway. Under complete Freund’s adjuvant (CFA)-induced inflammatory pain conditions, CB1 receptor expression at the messenger ribonucleic acid (mRNA) and protein levels is increased in the nerve fibers of skin of the hind paw and in primary afferent neurons located in the DRG in rats [79]. In a spinal nerve ligation injury model of neuropathic pain, CB1 receptor expression at the mRNA and protein levels was also increased in the DRG [77]. In chronic constriction injury (CCI) of the sciatic nerve, CB1 receptor expression was increased in the spinal cord [80]. Axotomy of the tibial branch of the sciatic nerve caused increased CB1 receptor expression in the thalamus contralateral to the injury [81]. In partial saphenous nerve ligation injury models of neuropathic pain in mice and rats, CB1 receptors were increased in the hind paw skin, DRG and spinal cord [82,83]. In the orofacial region, CCI of the infraorbital branch of the trigeminal nerve caused CB1 receptor expression to increase in the ipsilateral superficial laminae of the Vc as revealed by both western blot and immunohistochemistry analyses [84]. The increases in CB1 receptor expression may result in increased potency or efficacy of CB1 agonists or endocannabinoids under neuropathic pain conditions [61].

The presence of CB1 receptors in the peripheral nervous system and their increase under neuropathic and inflammatory pain conditions provides the scientific basis for targeting peripherally restricted CB1 receptors for neuropathic pain management. In a partial sciatic nerve ligation model of neuropathic pain, local intra-planter injection of a non-selective cannabinoid agonist attenuated mechanical hyperalgesia by activation of peripheral CB1 receptors [85]. In peripheral CB1 receptor knockout mice, the anti-hyperalgesic effects of systemically administered cannabinoids were almost completely lost following sciatic nerve injury and intra-plantar carrageenan injection, indicating the crucial role of peripheral cannabinoid receptors [52]. Studies have reported that local injection of cannabinoid agonists into inflamed tissue attenuated hyperalgesia and allodynia at doses that may produce minimal centrally-mediated side effects, suggesting peripheral CB1 receptor-mediated action [79,86].

### 2.2. Peripherally Active CB1 Receptor Agonists for the Treatment of Neuropathic Pain

Because peripherally restricted CB1 receptors are promising targets, peripherally active CB1 receptor agonists have been developed, and some have produced robust analgesic effects on neuropathic pain conditions with fewer CB1 receptor-mediated CNS side effects [63] (Table 1).

Spinal administration of the CB1 receptor-selective agonists ACEA attenuated mechanically-evoked responses of spinal neurons in a spinal nerve ligation neuropathic pain model, and this effect was blocked by a CB1-selective antagonist [87]. Systemic or local administration of this compound attenuated established mechanical allodynia in a chemotherapeutic agent (cisplatin)-induced neuropathic pain model in rats [88]. This compound showed no psychoactive effect at the dose used in that study [88]. Cannabinoid receptor agonist 13 (CRA13) is a CB1/CB2 dual agonist reported to produce strong anti-hyperalgesic effects in an animal model of neuropathic pain [89]. Both oral administration and local injection into the hind paw reversed established mechanical hyperalgesia [89]. At an oral dose of 3 mg/kg, the compound reversed mechanical hyperalgesia with rapid onset of action and long duration but without apparent cardiovascular and CB1 receptor-mediated CNS side effects [89]. A CB1-selective antagonist but not a CB2-selective antagonist inhibited the anti-hyperalgesic effects, indicating that peripheral CB1 receptors are mainly responsible for the anti-hyperalgesic action of the compound [89]. AZD1940, an orally active mixed CB1/CB2 receptor agonist, showed a CB1 receptor-dependent peripheral site of action for the analgesic effect in both inflammatory and neuropathic pain models in preclinical studies [63,93] without development of tolerance [90]. The brain uptake of this compound was low at anti-nociceptive doses in both rats and primates [90]. However, in clinical studies, the compound showed limited analgesic effects against capsaicin-induced pain and hyperalgesia and failed to attenuate surgical tooth extraction-induced post-operative pain in healthy humans with mild to moderate gastrointestinal and CNS side effects [94,95]. Systemic administration of another CB1/CB2 dual agonist, AZ11713908, reduced allodynia in a spinal nerve ligation injury model of neuropathic pain with minimal CB1 receptor-mediated CNS side effects [91]. After systemic administration, the brain uptake of this compound was low compared with that of a CNS-penetrant, mixed CB1 and CB2 receptor agonist, indicating its peripheral action. This compound also produced analgesic effects in a CFA-induced inflammatory pain model with fewer CNS side effects than the CNS-penetrant, mixed CB1 and CB2 receptor agonist [91]. The analgesic effect of this compound was absent in CB1 knockout mice but was present in CB2 knockout mice, confirming the CB1 receptor-mediated action of this compound [91]. Local intra-plantar application of this compound was also effective in producing robust analgesia [91]. Another orally bioavailable compound, LBP1, showed anti-allodynic and anti-hyperalgesic effects in a rat model of neuropathic pain without catalepsy [92], which is a common CB1 receptor-mediated side effect. This compound was found to have good water solubility and low brain uptake after systemic administration. A CB1 receptor antagonist blocked the anti-allodynic effects, indicating CB1-mediated action of this compound [92]. Systemic or oral administration of an indene-based compound named PrNMI attenuated sciatic nerve entrapment injury-induced mechanical allodynia [64]. The anti-allodynic effect was associated with few CB1-mediated CNS side effects (e.g., catalepsy, motor disturbance, hypothermia) and a low concentration of this compound in cerebrospinal fluid [64]. Inhibition of the analgesic effect of this compound by pretreatment with selective CB1 receptor but not CB2 receptor antagonists indicated the contribution of peripheral CB1 receptors [64].

## 3. Targeting CB2 Receptors for the Treatment of Neuropathic Pain

CB2 receptors were initially observed mainly in the immune tissues (e.g., spleen, tonsils) and cells (e.g., mast cells, lymphocytes) [47,96], where they appear to play a role in mediating the immunosuppressive effects of cannabinoids. Later, they were also found on neurons and nerve fibers [78,97,98,99,100]; however, in the brain, CB2 receptors are limited compared with the widespread distribution of CB1 receptors [98,101,102,103,104].

### 3.1. Modulation of CB2 Receptor Expression under Neuropathic Pain Conditions

Similar to CB1 receptors, CB2 receptor expression was also found to be modulated under inflammatory and neuropathic pain conditions at various sites of the pain processing pathway. CB2 receptor mRNA expression in the spinal cord was increased under neuropathic pain conditions induced by sciatic or spinal nerve ligation injury [103,104,105,106]. The mRNA level of this receptor was also increased in the DRG under neuropathic pain following spinal nerve ligation injury [103,104]. In a sciatic nerve CCI model of neuropathic pain, CB2 receptor expression at the protein level was increased in the spinal cord [107]. CB2 receptor expression in the hind paw skin, DRG and spinal cord was increased under neuropathic pain conditions following partial saphenous nerve ligation injury [82,83]. After sciatic nerve axotomy and in a spinal nerve ligation-induced neuropathic pain model, CB2 receptor expression at the protein level was increased in the DRG and in the primary afferent terminals in the spinal cord, which was not observed in CB2 receptor null mice [99]. Additionally, spinal nerve ligation injury increased CB2 receptor immunoreactivity in nerve sections proximal, but not distal, to the site of ligation [99]. CB2 receptor expression at the mRNA level was increased in the paw skin, DRG and spinal cord in a hind paw intra-plantar CFA-induced inflammatory pain model in rats [108]. The above studies suggest that CB2 receptors are upregulated at various sites of the pain pathway under inflammatory and neuropathic pain conditions.

CB2 receptors are also present in glial cells (e.g., microglia), which were observed to be upregulated following inflammation or nerve injury [109,110,111,112]. Microglia are important mediators for neuropathic pain development [113,114,115]. Various studies have reported that reactive microglia interact with neurons and contribute to the development of neuropathic pain [113,114,115]. In different models of neuropathic pain such as peripheral nerve injury, chemotherapy-induced neuropathic pain and chronic post-ischemia pain, CB2 receptor expression is increased in microglia [110,116,117]. In a sciatic nerve CCI model, CB2 receptor expression was observed in the spinal cord associated with injury-induced reactive microglia [105]. CB2 receptor expression in peripheral tissues was also reported to be increased under inflammatory conditions [118,119]. Under a local CFA injection-induced inflammatory pain condition, CB2 receptor expression at the mRNA and protein levels was increased in the inflamed skin tissue, and the receptors were mainly distributed in keratinocytes, macrophages and T-lymphocytes in the epidermis and dermis of the inflamed skin tissues [118]. CB2 receptors expressed in keratinocytes were increased under inflammatory or infectious conditions [118,119]. The presence of CB2 receptors in glial and inflammatory cells makes them attractive targets to reduce pain under inflammatory and neuropathic pain conditions [111,120].

Various selective CB2 receptor agonists have been reported to be effective in preclinical studies using models of inflammatory pain. Systemic administration of the CB2-selective agonists AM1241, GW405833, JWH133, and HU-308 attenuated thermal hyperalgesia/hypersensitivity/nocifensive behavior in intra-plantar carrageenan/CFA/formalin-induced inflammatory pain models [108,121,122,123]. Systemic administration of AM1241 inhibited substance P-induced plasma extravasation, suggesting inhibition of substance P-induced mast cell degranulation [124]. Local injection of this compound also attenuated hind paw carrageenan-induced edema and inflammatory hyperalgesia, which were reversed by co-administration of a CB2 receptor-selective antagonist [125]. Intra-plantar injection of JWH133 reduced innocuous and noxious mechanical stimuli-induced responses of spinal wide dynamic range (WDR) neurons in that model [121]. The action of this compound was often blocked by CB2 but not CB1 receptor-selective antagonists, suggesting peripheral CB2 receptor-mediated action. Intra-plantar injection of this compound also reduced carrageenan injection-induced expansion of peripheral receptive fields of WDR neurons [121]. Chronic administration of GW405833 was observed to reduce increased microglial and astrocyte expression in the spinal cord in a rat neuropathic pain model [126]. It also produced anti-hyperalgesic effects in incisional [127,128] and chronic inflammatory pain models [127]. The anti-hyperalgesic effect was not observed in CB2 knockout mice, suggesting a CB2 receptor-mediated mechanism of action for this compound [127,129]. Additionally, sedation and catalepsy were not observed for this compound at the anti-hyperalgesic dose used in the inflammatory pain model [127]. Some indole-based compounds observed to act as CB2 receptor inverse agonists and produced anti-nociceptive effects in the formalin-induced inflammatory pain model in mice [130].

### 3.2. Selective CB2 Receptor Agonists for the Treatment of Neuropathic Pain

Various selective CB2 receptor agonists were effective in attenuating neuropathic pain upon systemic or local application (intra-plantar, intra-spinal) in preclinical models of neuropathic pain (nerve-injury-induced, chemotherapeutic agent-induced) (Table 2).

A CB2 agonist, JWH015, upon local intra-plantar injection, attenuated mechanical allodynia and thermal hyperalgesia following sciatic nerve CCI in mice, which was antagonized by a CB2 receptor antagonist [131], suggesting peripheral CB2 receptor-mediated action. This compound also reduced mechanical allodynia in a streptozotocin-induced-diabetic neuropathy model in mice [132]. Upon local intra-plantar application, JWH133, another selective CB2 agonist, reduced innocuous and noxious mechanical stimuli-induced responses of WDR neurons in the spinal cord in a spinal nerve ligation injury model of neuropathic pain [121], suggesting a peripheral CB2 receptor-mediated action. The action of this compound was often blocked by CB2 but not CB1 receptor-selective antagonists [121]. Spinal administration of JWH133 attenuated mechanically-evoked responses of spinal neurons that were blocked by CB2-selective antagonists in the same type of neuropathic pain model, suggesting involvement of spinal CB2 receptors in the anti-nociceptive action [87]. Additionally, intra-spinal administration of this compound attenuated mechanical allodynia following partial sciatic nerve ligation injury in wild-type mice, whereas the action was absent in cannabinoid CB2 receptor knockout mice, confirming the involvement of CB2 receptors [144]. Local or systemic administration of JWH133 also attenuated mechanical allodynia in chemotherapeutic agent-induced neuropathic pain models without development of central side effects such as catalepsy, hypothermia and hypo-locomotion [88].

Systemic administration of AM1241 dose-dependently reversed tactile and thermal hypersensitivity in a rat spinal nerve ligation model of neuropathic pain [101,138]. The anti-nociceptive action of this compound was inhibited by a CB2 but not a CB1 receptor antagonist and was retained in CB1 knockout mice, confirming CB2-mediated action of this compound [101]. AM1241 did not elicit motor impairment, hypothermia and catalepsy (which are considered to be CB1-mediated central side effects) [150]. Intra-spinal administration of this compound reversed the expression of p38 mitogen-activated kinase (MAPK), interleukin-1 beta (IL-1β) interleukin-10 (IL-10), MAGL and astrocytes in the spinal cord, resulting in levels similar to those in non-neuropathic controls [138,139]. Systemic administration of AM1241 attenuated mechanical allodynia in chemotherapeutic agent (vincristine or cisplatin)-induced neuropathic pain models without development of catalepsy [134], and the action was antagonized by a CB2 receptor antagonist [133,134]. In a streptozotocin-induced diabetic neuropathy model, AM1241 reduced mechanical allodynia [135,136], and upon systemic administration, this compound also reduced hind paw incision-induced post-surgical pain, which was antagonized by a CB2 receptor antagonist [128]. Systemic administration of AM1241 also reduced thermal and mechanical hyperalgesia evoked by intra-plantar administration of capsaicin, which was antagonized by a CB2 but not a CB1 receptor antagonist, indicating the involvement of CB2 receptors in the anti-nociceptive action of the compound [151]. AM1241 was used in a drug self-administration approach where the animal administered the drug by itself, and neuropathic animals self-administered the compound to attenuate mechanical allodynia [137].

AM1710, another CB2-selective agonist, attenuated mechanical and cold allodynia upon acute or chronic systemic administration in a rat model of cisplatin and paclitaxel-induced neuropathy, and the action was antagonized by a CB2 receptor antagonist [145,147]. The anti-allodynic effect of systemic administration of this compound was absent in CB2-knockour mice, indicating that CB2 receptors mediated the action of this compound [147]. AM1710 also reduced pro-inflammatory cytokines and chemokines (tumor necrosis factor-α (TNF-α) and monocyte chemoattractant protein-1 (MCP-1)) in the spinal cord upon acute or chronic systemic administration [147]. This compound did not elicit hypothermia, hypo-locomotion or ataxia (cannabimimetic side effects) [147,152]. Additionally, chronic systemic application of AM1710 prevented the development of mechanical and cold allodynia in a paclitaxel-induced neuropathic pain model without development of tolerance, physical withdrawal, hypothermia, or motor dysfunctions [147]. Chronic subcutaneous application of this compound prevented the development of mechanical and cold allodynia in a paclitaxel-induced neuropathic pain model [146]. Intrathecal application of AM1710 in the spinal cord also attenuated mechanical and cold allodynia in a paclitaxel-induced neuropathic pain model [147]. The anti-allodynic efficacy of intrathecal administration of AM1710 was absent in CB2 knockout mice [147], indicating the CB2 receptor-mediated action of this compound [147]. AM1714, another variety of the AM series, attenuated mechanical allodynia upon systemic administration in a rat model of paclitaxel-induced neuropathy [134].

Repeated administration of another CB2-selective agonist, NESS400, produced anti-nociceptive effects in the spared nerve injury model of neuropathic pain in mice [143]. Spared nerve injury caused activation of microglia, and astrocytes in the dorsal horn of the spinal cord and treatment with this compound significantly reduced the number of hypertrophic microglia and astrogliosis [143]. 

In partial sciatic nerve ligation [127,129] and sciatic nerve CCI injury [140] models of neuropathic pain, systemic administration of GW405833 (a potent, selective CB2 agonist) elicited anti-allodynic effects [127,129,140]. At a low effective anti-allodynic dose, it did not elicit sedation and catalepsy [127], but at a high dose, it produced motor impairment [129]. Chronic administration of this compound reduced mechanical allodynia in a modified spinal nerve ligation injury model of neuropathic pain associated with reduced expression of microglia and astrocytes in the spinal cord [126]. 

The CB2-selective agonists MDA7 and MDA19 were investigated in spinal nerve ligation injury and paclitaxel-induced neuropathic pain models [117,141,142,153]. Systemic administration of these compounds attenuated mechanical allodynia without affecting locomotor activity [141,142]. The actions of these compounds were inhibited by CB2 receptor antagonists [117,141,142], and in paclitaxel-induced neuropathic mouse models, the action was absent in CB2 knockout mice, confirming its CB2-mediated action [117,142]. MDA7 was effective in preventing the development of mechanical allodynia upon chronic systemic administration in paclitaxel-induced neuropathic models [117]. Systemic administration of this compound reduced the paclitaxel-induced increase in expression of microglia and astrocytes in the spinal cord and attenuated the paclitaxel-induced neuro-inflammatory response as evidenced by downregulation of Toll-like receptor 2 (TLR2), extracellular signal regulated kinase 1/2 (ERK1/2) and CB2 receptor expression in the spinal cord [117]. MDA7 also decreased the release of pro-inflammatory mediators (IL-1β and TNF-α) from lipopolysaccharide-stimulated cultured astrocyte cells in vitro [117]. 

Along with the synthetic agents described above, the natural CB2 agonist β-caryophyllene (BCP, a constituent of essential oils) reduced established mechanical allodynia upon oral administration in a paclitaxel-induced neuropathic pain model [108]. Chronic oral administration of this agonist along with paclitaxel attenuated the development of neuropathic pain [108]. This chronic treatment reduced the paclitaxel-induced spinal cord increases in reactive microglia, pro-inflammatory cytokine IL-1β release, p38 MAPK and nuclear factor kappa-light-chain-enhancer of activated B cells (NF-κB) [108], suggesting an anti-inflammatory action of this CB2 agonist. Chronic oral administration of BCP also produced anti-allodynic and anti-hyperalgesic effects in partial sciatic nerve injury-induced neuropathic mice, which were absent in CB2 knockout mice, confirming CB2 receptor-mediated action [106]. This agonist did not elicit motor disturbance, hypothermia or catalepsy after chronic oral administration in mice [106]. It also reduced the nerve injury-induced increases in expression of microglia and astrocytes in the spinal cord and produced an anti-allodynic effect in the late phase of a formalin-induced inflammatory pain model in mice [106].

The above studies demonstrated the contribution of CB2 receptors located in neurons, glial cells and inflammatory cells to the anti-nociceptive action of selective CB2 receptor agonists in inflammatory and neuropathic pain models. Activation of CB2 receptors by agonists inhibited sensory nerve activity in animal models of acute and chronic pain, suggesting direct action on sensory neurons [87,121,129,154,155]. Additionally, nerve/tissue injury can recruit glial cells to various sites of pain pathways and inflammatory cells to the injury site, which release various inflammatory mediators to produce nerve sensitization. CB2 receptor agonists can reduce these inflammatory mediators and decrease the sensitization of the nerves [111,120].

## 4. Targeting Endocannabinoids for the Treatment of Neuropathic Pain

Another potential alternative approach for neuropathic pain control with reduced risk of side effects is targeting endocannabinoids [19,20,67,68,69]. Natural endocannabinoids are lipids. Among endocannabinoids, 2-AG and AEA are extensively studied in various types of research. AEA belongs to the N-acylethanolamine (NAE) and 2-AG belongs to the 2-acylglycerol (2-AcG) families of lipids [35,156,157]. AEA is one of the least abundant NAEs, while 2-AG is among the most abundant 2-AcGs [158]. In the brain, the 2-AG level is higher than that of AEA [159]. AEA has been observed to be a partial agonist for both CB1 and CB2 receptors, while 2-AG binds to both receptors with the same affinity and exhibits higher potency in activating CB1 and CB2 receptors than AEA [69,157,160]. Unlike well-studied hydrophilic neurotransmitters (e.g., glutamate, γ-amino butyric acid (GABA), acetylcholine), endocannabinoids are not stored in synaptic vesicles; instead, they appear to be biosynthesized and released by neurons at the moment of their intended action, which is often referred to as “on-demand” production [35]. The metabolic pathways for endocannabinoids are complex and have reviewed in detail [31,32,33,34,35]. Evidence suggests that endocannabinoids are synthesized from postsynaptic neurons upon activation, rather than being pre-stored, and act on presynaptic neurons to inhibit the release of various neurotransmitters, a phenomenon known as retrograde suppression of synaptic transmission [161,162,163]. After their release, both AEA and 2-AG are cleared through cellular uptake facilitated by putative endocannabinoid transporters followed by intracellular enzymatic hydrolysis. AEA is degraded primarily by the FAAH enzyme [31,164], while 2-AG is degraded primarily by MAGL [164,165]. Other enzymes may also be involved in degradation. AEA and 2-AG can also be oxidized/degraded by cyclooxygenase (COX) enzymes [20,166]. FAAH may also contribute to 2-AG degradation [165]. Because of their rapid degradation, endocannabinoids have relatively short durations of action [165]. 

Endocannabinoids in the body are anti-nociceptive and may participate tonically in the intrinsic control of pain initiation [167,168]. The anti-nociceptive action of endocannabinoids has been observed in various studies [167,168,169]. Inhibition of peripheral or central CB1 receptors leads to hyperalgesia in normal animals, indicating tonic action of endocannabinoids to control pain [20,167,168]. Endocannabinoid deficiency may also contribute in the development of chronic painful disorders (e.g., migraine) [170,171].

### 4.1. Modulation of Endocannabinoids under Neuropathic Pain Conditions

Various studies have reported that endocannabinoid levels are modulated at different sites of the pain pathway under inflammatory and neuropathic pain conditions [19,21,172]. The level varies with the nature of the pathological condition. Endocannabinoids may be increased because of the response of an endogenous neuroprotective mechanism to a pathological condition [19,21]. The recruitment of immune cells at the site of nerve injury/inflammation may provide a further source of endocannabinoid synthesis and metabolism [19,21,173,174,175]. Endocannabinoids can be synthesized from reactive glial cells [176,177,178], which are observed to be increased under inflammatory and neuropathic pain conditions [113,179].

Increased endocannabinoid levels are observed in the CNS under neuropathic and inflammatory pain conditions [180,181,182]. Both AEA and 2-AG are increased in the descending pain modulating network (e.g., PAG, RVM, and dorsal raphe nucleus) and spinal cord under neuropathic pain induced by CCI of the sciatic nerve [180,181]. These compounds were also increased in the spinal cord in a spinal nerve ligation injury model of neuropathic pain [181] and in a cisplatin-induced neuropathic pain model [183]. The FAAH mRNA level was also increased in the DRG in a cisplatin-induced neuropathic pain model [183]. Another study showed that AEA but not 2AG was increased in the dorsal raphe nucleus (which contains serotonergic neurons) following CCI of the sciatic nerve [184]. Intra-plantar formalin injection also increased the AEA level in the PAG [185].

Endocannabinoids are active not only in the CNS but also in the PNS, and their levels are modulated in peripheral areas under inflammatory and neuropathic pain conditions. In a spinal nerve ligation injury model of neuropathic pain, both AEA and 2-AG were significantly increased in the DRG, which contains peripheral afferent neurons [77]. Local subcutaneous injection of exogenous 2-AG and AEA produced anti-nociceptive effects in an inflammatory pain model induced by intra-plantar formalin injection, indicating peripheral action of endocannabinoids [167,186]. Additionally, local injection of AEA was not accompanied by central side effects and exerted a 100-times greater anti-nociceptive effect than systemic administration, confirming the peripheral site of action [167]. A study using a spinal nerve ligation injury model of neuropathic pain reported that AEA and *N*-oleoylethanolamine but not 2-AG were increased in the hind paw ipsilateral to the injury [187]. In humans with inflammatory conditions of the colon, the AEA but not the 2-AG level was increased [188]. In a carrageenan-induced inflammatory pain model, intra-plantar injection of AEA reversed the peripheral mechanically-evoked responses of spinal neurons, and the action was blocked by both CB1 and CB2 receptor antagonists, suggesting contribution of peripherally-expressed CB1 and CB2 receptors to the AEA-induced anti-nociceptive response [189]. AEA can also activate TRPV1 channels [40]. TRPV1 is implicated in processing of various types of pain including neuropathic and inflammatory pain [182,190,191,192]. TRPV1 channels are easily desensitized by TRPV1 agonists (e.g., capsaicin) [193]. Desensitization of TRPV1 by intra-planter AEA injection may also contribute to the local action of AEA. Systemic administration of AEA was also observed to produce anti-nociceptive action in a number of acute and inflammatory pain models, albeit with less efficacy than synthetic cannabinoid receptor agonists [167,169,194,195].

### 4.2. Endocannabinoid Degradation Enzyme Inhibitors for the Treatment of Neuropathic Pain

Because endocannabinoids are rapidly degraded by their degrading enzymes, compounds that can inhibit those enzymes have been developed. These compounds can increase the endocannabinoid levels at the sites where they are synthesized on demand by preventing their degradation. The localized increase of endocannabinoids is thought to produce localized action compared with the global action of exogenously applied cannabinoids [19,21,196,197,198]. Various preclinical studies have reported that these compounds produced anti-nociceptive effects with fewer or no side effects in diverse models of inflammatory and neuropathic pain [19,20,21,67,68,69].

#### 4.2.1. FAAH Inhibitors

Because FAAH is the main enzyme responsible for AEA degradation [164], various FAAH inhibitors have been developed and tested in preclinical animal models (Table 3).

A FAAH inhibitor, OL135, reduced mechanical allodynia and acetone-induced cold allodynia in a sciatic nerve CCI model of neuropathic pain upon systemic administration [199]. Brain and spinal cord AEA levels increased after administration of the compound [199]. The anti-nociceptive actions of OL135 were antagonized by both CB1 and CB2 receptor antagonists, indicating that both CB1 and CB2 receptors were activated by the elevated AEA level [199].

Systemic, oral, intra-spinal and intra-plantar injection of a selective FAAH inhibitor, URB597, was found to produce anti-nociceptive effects in neuropathic pain models. In the sciatic nerve CCI model of neuropathic pain, systemic administration of URB597 reduced mechanical allodynia and acetone-induced cold allodynia, and this action was not observed in FAAH knockout mice [199]. The anti-nociceptive actions of this compound were antagonized by both CB1 and CB2 receptor antagonists [199]. URB597 also increased brain and spinal cord AEA levels [199]. Chronic oral administration of this compound dose-dependently attenuated the nocifensive behavior to thermal and mechanical stimuli in a sciatic nerve CCI model of neuropathic pain [202], and the effect was inhibited by a CB1 receptor antagonist. Oral dosing of this compound reduced brain FAAH activity and increased the spinal cord AEA level [202], indicating the efficacy of this compound to reduce degradation of AEA. URB597 also reduced plasma extravasation in the paws of CCI mice [202]. Additionally, this compound produced anti-nociceptive effects upon intra-spinal administration in the same model of neuropathic pain in which the effects were antagonized by CB1 or CB1/TRPV1 receptor antagonists, depending on the dose of the compound [203]. The effect of a low dose was antagonized by a CB1 receptor antagonist, whereas the effect of a high dose was antagonized by a TRPV1 antagonist [204], suggesting that the anti-nociceptive effect of a high dose of the compound could be attributed to the desensitization of TRPV1 channels by a high AEA level [204]. Local injection of URB597 in the hind paw also reduced mechanical allodynia and thermal hyperalgesia following partial sciatic nerve ligation injury, which was antagonized by both CB1 and CB2 receptor antagonists in rats [206]; however, in mice, the action was antagonized by a CB1 receptor antagonist, and the action was absent in CB1 but not CB2 knockout mice [205], indicating species differences in the involvement of these receptors. This compound was found to reduce mechanical allodynia and thermal hyperalgesia upon systemic administration in an intra-plantar CFA injection model of inflammatory pain in rats [218]. Co-administration of CB1 and CB2 receptor antagonists completely reversed these effects, indicating that both CB1 and CB2 receptors mediated this action in rats [218]. Acute systemic administration of URB597 reduced mechanical and cold allodynia in a cisplatin-induced neuropathic pain model [183], and the effect was antagonized by CB1, CB2, and TRPV1 receptor antagonists. Upon chronic systemic administration, URB597 prevented the development of paclitaxel-induced neuropathic pain and reduced previously established allodynia in this model [210].

Oral administration of another FAAH inhibitor, PF3845, reduced allodynia and increased the brain AEA level in an intra-plantar CFA-induced inflammatory pain model in rats [219]. A combination of CB1 and CB2 receptor antagonists blocked the anti-allodynic effect of this compound, indicating involvement of both CB1 and CB2 receptors [219]. PF3845 also reduced mechanical and cold allodynia following sciatic nerve CCI in mice [214,215] without development of tolerance to its anti-nociceptive effects and desensitization of brain CB1 receptors [214]. Additionally, systemic administration of PF3845 reduced pain in an intra-plantar carrageenan-induced inflammatory pain model [220]. Another FAAH inhibitor of the PF series, PF04457845, was observed to be highly anti-nociceptive after oral administration in a CFA-induced inflammatory pain model and in a monosodium iodoacetate-induced non-inflammatory pain model in animals [198]. Animals treated with this compound did not show catalepsy, hypo-motility or a change in body temperature [198].

Some tetrazole-based compounds (e.g., LY2183240) may act as both AEA reuptake inhibitor, and FAAH inhibitor produced anti-nociceptive effects in the formalin-induced inflammatory pain model partly through indirect activation of cannabinoid receptors [221].

Oral administration of a reversible FAAH inhibitor, ST4070, showed strong anti-allodynic effects in neuropathic pain models induced by sciatic nerve CCI, the chemotherapeutic agent vincristine and the diabetes-producing agent streptozotocin [200]. The anti-allodynic effects of this compound in a sciatic nerve CCI-induced neuropathic pain model were attenuated by pretreatment with CB1 and CB2 receptor antagonists and by a selective PPARα antagonist [200].

A brain impermeant FAAH inhibitor, URB937, has recently been developed and found to be effective in various models of neuropathic and inflammatory pain [216,217]. This inhibitor has a limited ability to cross the blood–brain barrier and increases the AEA level in peripheral tissues [216]. Systemic or oral administration of this compound attenuated mechanical allodynia and thermal hyperalgesia in a mouse model of neuropathic pain [216,217], which was antagonized by a CB1 but not a CB2 receptor antagonist [216]. URB937 was also effective in reducing mechanical allodynia and thermal hyperalgesia in an intra-plantar carrageenan injection-induced inflammatory pain model [216,217]. Interestingly, this compound was observed to be more effective than other standard analgesic and anti-inflammatory drugs (such as indomethacin, gabapentin, and dexamethasone) [217]. It was also more effective than the brain-permeant FAAH inhibitors URB597 and PF04457845 in an intra-plantar CFA injection-induced inflammatory pain model [217]. In a chemotherapeutic agent (cisplatin)-induced neuropathic pain model, systemic administration of URB937 reversed mechanical and cold allodynia [183]. The anti-allodynic effect of this compound was antagonized by CB1, CB2, and TRPV1 receptor antagonists [183]. Chronic systemic administration of URB937 prevented the development of chemotherapeutic agent (paclitaxel)-induced neuropathic pain and reduced previously established allodynia in this model [210].

##### Dual FAAH and TRPV1 Inhibitors

Because an increase in AEA by an FAAH inhibitor can also act on TRPV1, which has been implicated in the development of neuropathic pain, a dual FAAH and TRPV1 inhibitor, AA-5-HT (N-arachidonoyl-serotonin) has been tested in a neuropathic pain model [207,208]. The compound produced enhanced anti-nociceptive effects in neuropathic pain models in rats compared with separate use of an FAAH inhibitor (PF3845) or a TRPV1 inhibitor (iodoresiniferatoxin) [207].

##### Combining FAAH Inhibitors with NSAIDs or COX2 Inhibitors

FAAH inhibitors were also combined with non-steroidal anti-inflammatory drugs (NSAIDs) (e.g., indomethacin) or COX2 inhibitors (e.g., diclofenac) [213,217,222]. These combinations were found to produce enhanced anti-nociceptive effects in neuropathic and inflammatory pain models [213,217] and reduce NSAID-induced gastrointestinal problems [217]. The combination of subthreshold doses of an FAAH inhibitor, PF3845, and a COX2 inhibitor, diclofenac, produced enhanced anti-allodynic effects in neuropathic and inflammatory pain models [213] compared with separate use of the FAAH inhibitor or COX2 inhibitor. The combination also increased the brain AEA level and decreased the brain prostaglandin level [213]. The combination of a brain-impermeant FAAH inhibitor, URB937, with indomethacin was observed to produce a synergistic anti-nociceptive effect in sciatic nerve CCI-induced neuropathic and carrageenan injection-induced inflammatory pain models [217]. This combination also reduced indomethacin-induced gastric lesions [217].

#### 4.2.2. MAGL Inhibitors

MAGL inhibitors were developed more recently, and relatively few studies have examined MAGL inhibitors in neuropathic pain models (Table 4) compared with FAAH inhibitors.

Initial compounds developed for inhibition of MAGL (e.g., *N*-arachidonyl maleimide, methylarachidonylfluorophosphonate) were found to be poorly selective for MAGL and had off-target effects [229,230,231,232]. A compound named URB602 was observed to be nonselective for MAGL in an in vitro study, but in in vivo studies, systemic [233], local [233], or intracerebral [234] administration of this compound increased the 2-AG level without increasing the AEA level. Intra-plantar URB602 injection reduced the early and late phases of formalin-induced pain [233], which was antagonized by CB1 and CB2 receptor antagonists.

Recently developed MAGL inhibitors, including JZL184, KML29, and MJN110 were observed to have more selectivity for MAGL [197,211,224]. Acute systemic administration of JZL184 was observed to be anti-nociceptive in nerve injury-induced [199,201,212,214,215,223,224,225,226] and chemotherapeutic agent (e.g., cisplatin, paclitaxel)-induced neuropathic pain models [183,209,227]. Systemic administration of JZl184 attenuated mechanical and acetone-induced cold allodynia in a sciatic nerve CCI model of neuropathic pain that was antagonized by CB1 but not CB2 receptor antagonists [199]. Brain and spinal cord 2-AG levels were also increased after JZL184 administration [199]. Repeated systemic administration of both high and low doses of JZL184 produced anti-nociceptive effects in a sciatic nerve CCI model of neuropathic pain; however, the high but not the low dose caused downregulation and desensitization of brain CB1 receptors, decrements in endocannabinoid-dependent synaptic plasticity and development of tolerance to its anti-nociceptive effects [214,220,223], suggesting that a great increase in the brain 2-AG level by a high dose might cause these problems. In cisplatin- or paclitaxel-induced neuropathy models, systemic administration of JZL184 attenuated mechanical and cold allodynia, which was antagonized by both CB1 and CB2 receptor antagonists [183,227]. The anti-allodynic effect was not observed in CB1 and CB2 knockout mice, confirming its CB1 and CB2 receptor-mediated action [227]. Repeated systemic administration of a threshold dose of this compound completely reversed paclitaxel-induced allodynia without development of tolerance, while a high dose induced tolerance to its anti-nociceptive effect [227]. Chronic local intra-plantar injection of JZL184 was also effective in reducing mechanical hyperalgesia in a cisplatin-induced neuropathic pain model [228].

JZL184 was also effective in attenuating pain in intra-plantar carrageenan [220] and formalin [235] injection-induced inflammatory pain models. Systemic administration of JZL184 reduced pain in an intra-plantar carrageenan-induced inflammatory pain model, and the action was antagonized by both CB1 and CB2 receptor antagonists [220]. Inflammation-induced expansion of the receptive fields of spinal WDR neurons in intra-plantar carrageenan injected rats was abolished by intra-spinal administration of JZL184 [236]. Local intra-paw administration of JZl184 was observed to have potent anti-nociceptive actions on intra-plantar formalin-induced pain, suggesting that increased paw skin 2-AG accumulation caused by this MAGL inhibitor could be attributed to its anti-nociceptive actions [235]. Intra-paw administration of 2-AG also showed anti-nociceptive actions; this confirmed the local action of 2-AG. This compound also suppressed capsaicin-induced behavioral sensitization [237].

Systemic administration of another selective MAGL inhibitor, KML29, attenuated mechanical and cold allodynia in a sciatic nerve injury model of neuropathic pain [224,225] without elicitation of cannabimimetic side effects such as catalepsy, hypothermia and hypo-motility [224]. Acute or repeated administration of this compound increased the brain 2-AG level without elevating the brain AEA level, suggesting its specificity for MAGL. KML29 also reduced allodynia and paw edema in an intra-plantar carrageenan injection-induced inflammatory pain model [224]; however, repeated administration of this compound induced tolerance to its anti-nociceptive effects [224]. The anti-allodynic effect in the inflammatory model was mediated by both CB1 and CB2 receptors, and the anti-allodynic effect in the neuropathic pain model was mediated by CB1 receptors [224].

Another recently developed selective MAGL inhibitor, named MJN110, was observed to be highly potent in attenuating mechanical allodynia and thermal hyperalgesia in a sciatic nerve CCI model of neuropathic pain in mice [226]. When the potency of JZL184 and MJN110 was compared, systemic administration of MJN110 was observed to be 42-fold more potent than administration of JZL184 (ED_50_ values of 0.43 and 17.8 for MJN110 and JZL184, respectively) in attenuating mechanical allodynia and thermal hyperalgesia in this model [226]. Both JZL184 and MJN110 increased brain 2-AG levels without altering AEA levels, suggesting their selectivity for MAGL [226]. Although JZL184 produced hypo-motility (a CB1 receptor-mediated CNS side effect), MJN110 did not produce hypo-motility, catalepsy or hypothermia; instead, MJN110 was observed to increase locomotor activity [226]. This compound also alleviated mechanical allodynia in a rat model of diabetic neuropathy [211]. In a chemotherapeutic agent (paclitaxel)-induced neuropathy model in mice, systemic administration of MJN110 reversed mechanical allodynia, and the effect was mediated by both CB1 and CB2 receptors. MJN110 was more potent than JZL184 in reversing allodynia [227]. MJN110 also reduced the paclitaxel-mediated increases in expression of chemokines, MCP-1 in the DRG and spinal dorsal horn and phospho-p38 MAPK in the DRG, suggesting its anti-inflammatory action [227].

##### FAAH Inhibitors Combined with MAGL Inhibitors

The combination of FAAH and MAGL inhibitors has also been used in preclinical models of neuropathic and inflammatory pain [206,212]. A combination of a high dose of an FAAH inhibitor, PF3845, and a low dose of an MAGL inhibitor, JZL184, showed anti-nociceptive action in sciatic nerve CCI-induced neuropathic and carrageenan-induced inflammatory pain models [212]. This combination, upon systemic administration, produced greater anti-nociceptive effects than those of the single inhibitors without common cannabimimetic side effects (e.g., catalepsy, hypo-motility, hypothermia) [212]. The combination caused a >10-fold increase in the brain AEA level and a 2- to 3-fold increase in brain 2-AG levels [212]. Repeated administration of this combination in the inflammatory pain model did not induce tolerance to its anti-allodynic actions [212].

##### Dual MAGL and FAAH Inhibitors

Dual MAGL and FAAH inhibitors have been developed and tested in preclinical inflammatory and neuropathic pain models [201,238]. In a sciatic nerve CCI model of neuropathic pain, JZL195 {4-Nitrophenyl 4-(3-phenoxybenzyl)-1-piperazinecarboxylate} (a dual MAGL and FAAH inhibitor) attenuated mechanical allodynia and acetone-induced cold allodynia [201]. The anti-nociceptive efficacy of this compound was greater than that of JZL184 (an MAGL inhibitor) or URB597 (an FAAH inhibitor). At a low but effective anti-nociceptive dose, the compound did not produce CB1 receptor-mediated central side effects, but at a high dose, it produced side effects that were greater than those produced by JZL184 or URB597 alone [201]. This compound was also effective in reducing mechanical allodynia and thermal hyperalgesia in an intra-plantar CFA injection-induced inflammatory pain model in mice at doses lower than those that produce side effects [239]. The action of this compound was mediated by both CB1 and CB2 receptors [239]. JZL195 was also effective in producing anti-nociceptive effects in acute thermal, visceral and inflammatory pain models [240,241].

Another dual MAGL-FAAH inhibitor, SA-57 [4-[2-(4-chlorophenyl)ethyl]-1-piperidinecarboxylic acid 2-(methylamino)-2-oxoethyl ester], was tested in sciatic nerve CCI-induced neuropathic pain and carrageenan inflammatory pain models in mice and found to be effective in reducing mechanical allodynia upon systemic administration in the both models [238]. The anti-allodynic effect of this compound was not observed in CB1 and CB2 knockout mice following carrageenan injection in the paw, indicating that both receptors were required for the effect in the inflammatory pain model. However, carrageenan injection induced paw edema, which was retained after systemic administration of this compound in CB1 but not in CB2 knockout mice, indicating that reduction of inflammatory edema by SA-57 required CB2 receptors [238]. Administration of this compound at anti-nociceptive doses produced cannabimimetic side effects including catalepsy, hypothermia and impaired locomotion [238].

##### MAGL Inhibitors Combined with Opioids

Opioids are used for the treatment of neuropathic pain; however, they have a wide range of side effects, and their abuse rate is also high [16,17]. To overcome these problems, combinations of opioids and endocannabinoid degrading enzyme inhibitors have been tested in preclinical models of neuropathic pain. The combination of MJN110 and morphine (an opioid), upon systemic administration, reversed mechanical allodynia and thermal hyperalgesia in a sciatic nerve CCI model of neuropathic pain in mice [242]. Acute systemic administration of this combination did not reduce gastric motility (a common side effect of morphine) or produce subjective cannabimimetic effects (side effects of CB1 receptor agonists). Repeated dosing of this combination was also effective in attenuating allodynia and hyperalgesia without development of tolerance (a side effect that can be produced by extended use of opioids), indicating that addition of an MAGL inhibitor produces opioid-sparing events [242].

The dual MAGL-FAAH inhibitor SA-57 was combined with morphine at their threshold doses, and this combination was found to be effective in completely reversing sciatic nerve CCI-induced allodynia. Furthermore, it did not produce tolerance following repeated administration [238]. The combination did not elicit hypo-motility, catalepsy, or hypothermia, indicating that the increase in the brain endocannabinoid level may be minimal, thus limiting the side effects. Interestingly, this compound decreased heroin seeking behavior in mice, indicating a reduction of opioid abuse [238].

The above findings suggest that combining opioids with endocannabinoid degrading enzyme inhibitors has a potential benefit in reducing the effective doses of opioids needed for pain control (opioid-sparing) and reducing development of opioid- or cannabinoid-mediated side effects and tolerance.

##### Combining MAGL Inhibitors with COX2 Inhibitors

An MAGL inhibitor was also combined with a COX2 inhibitor and found to produce an improved anti-nociceptive effect in a neuropathic pain model [225]. JZL184 or KML29 was combined with diclofenac, and the combination produced synergistic anti-allodynic effects upon systemic administration in a sciatic nerve CCI model of neuropathic pain [225]. The anti-allodynic effects of the combination were antagonized by CB1 but not CB2 receptor antagonists, indicating involvement of CB1 receptor-mediated action [225]. Systemic administration of the combination also reduced the spinal cord prostaglandin level [225].

## 5. Potential of Endocannabinoid Enzyme Inhibitors and Cannabinoid Receptor Agonists for the Treatment of NOP

Although many studies have been conducted to examine the efficacy and mechanisms of action of cannabinoids and endocannabinoids in animal models of neuropathic and inflammatory pain conditions in parts of the body outside the orofacial region, studies using orofacial pain models are rare [243,244].

### 5.1. Modulation of Cannabinoid Receptors and Endocannabinoids in the Orofacial Region under Inflammatory and Neuropathic Pain Conditions

In the orofacial region, similar to other body regions, cannabinoid receptors and endocannabinoids have been observed to be modulated under inflammatory and neuropathic pain conditions. In a rat model of NOP induced by CCI of the infraorbital nerve (a branch of the trigeminal nerve), CB1 receptor expression at the protein level increased within the superficial laminae of the brainstem Vc [84]. In humans, CB1 and CB2 receptor expression at the mRNA and protein levels was increased in gingival tissues under inflammatory conditions (in gingivitis and periodontitis) [245]. Increased expression of CB2 but not CB1 receptors was observed in tongue epithelial cells in patients with burning mouth syndrome [246]. In migraine patients, AEA and 2-AG were observed to be downregulated, and FAAH was upregulated [247,248]. In a nitroglycerin-induced migraine pain model in rats, FAAH activity was increased in the medulla and hypothalamus, and the activity of both FAAH and MAGL was increased in the mesencephalon [249].

### 5.2. Modulation of Orofacial Neuronal Activity by Cannabinoids and Endocannabinoids

Studies have reported the modulation of orofacial neuronal activity by cannabinoids or endocannabinoids. Perfusion of brainstem slices with a synthetic cannabinoid, WIN 55,212-2, or an endocannabinoid, AEA, hyperpolarized neurons located in the Vc and reduced the amplitude of excitatory postsynaptic potentials (EPSPs) or currents evoked by stimulating the mandibular nerve (a branch of the trigeminal nerve) [250], suggesting the inhibition of primary afferent glutamatergic transmission by cannabinoids or endocannabinoids [250]. An N-type Ca^2+^ channel blocker reversed the action of WIN 55,212-2, suggesting inhibition of N-type Ca^2+^ channels by the compound. The reduction of EPSP amplitude by WIN 55,212-2 was abolished by a CB1 receptor antagonist, indicating involvement of CB1 receptors [250]. Local application of WIN 55,212-2 in the Vc reduced the activity of WDR neurons in response to transcutaneous electrical stimulation of the face, which was antagonized by a CB1 receptor antagonist [251]. WIN 55,212-2 also potentiated GABA-evoked inward currents in neurons freshly isolated from the rat TG, which was antagonized by a CB1 receptor antagonist [252]. This compound also inhibited 5-hydroxytryptamine receptor 3 (5-HT_3_)-activated currents in cultured rat TG neurons [253] without involving CB1 or CB2 receptors. Additionally, WIN 55,212-2 was found to inhibit capsaicin-induced current in cultured rat TG neurons [254]. Application of AEA in cultured rat TG neurons led to release of a neuropeptide (calcitonin gene-related peptide), which was inhibited by a TRPV1 antagonist, suggesting activation of TRPV1 receptors by AEA [255].

### 5.3. Evidence of the Anti-Nociceptive Effects of Endocannabinoid Degrading Enzyme Inhibitors and Cannabinoid Receptor Agonists in NOP Models

Studies have investigated the efficacy of cannabinoid receptor agonists and endocannabinoid degrading enzyme inhibitors in animal models of NOP (Table 5).

Cannabinoids and endocannabinoids were found to be anti-nociceptive in migraine pain and to inhibit trigeminovascular neurons that may be activated in migraine pain [259,260,261]. In a nitroglycerin-induced migraine pain model, systemic administration of FAAH inhibitors, URB597 or PF3845, attenuated nitroglycerin-induced mechanical hyperalgesia in mice, indicating elevation of the AEA level by the FAAH inhibitors and a reduction of migraine pain [248]. The action of both FAAH inhibitors was antagonized by a CB1 receptor antagonist. Administration of FAAH inhibitors also reduced nitroglycerin-induced c-Fos (a marker of neuronal activation) expression in the brainstem trigeminal nuclei, which was blocked by a CB1 receptor antagonist [248]. Additionally, knockout of FAAH but not MAGL attenuated nitroglycerin-induced mechanical hyperalgesia, indicating a crucial role of AEA [248] in attenuating pain. Systemic administration of exogenous AEA attenuated intra-plantar formalin-induced nocifensive behavior and nitroglycerin-induced c-Fos expression in the Vc [249]. In another study, the brain-impermeant FAAH inhibitor URB937 was observed to be anti-nociceptive in a nitroglycerin-induced migraine pain model, suggesting that elevation of AEA in the peripheral nerves and tissues can attenuate migraine pain [257]. Systemic administration of this compound increased the tail-flick latency to radiant heat and attenuated nociceptive behavior (number of flinches and shakes) induced by formalin injection into the hind paw and upper lip [257]. Administration of this compound also reduced c-Fos expression in the Vc [257]. The selective CB2 agonist AM1241 was also found to be effective in increasing tail-flick latency in response to a high intensity light beam and in reducing nociceptive behavior induced by formalin injection into the hind paw in a nitroglycerin-induced migraine pain model in rats [256]. The synthetic cannabinoid receptor agonist WIN 55,212-2 reduced Vc neuronal activity with regard to receiving convergent input from the dura mater and face. A-fiber and C-fiber inputs, as well as spontaneous firing, were inhibited by the compound [260]. The anti-nociceptive effect was reversed by CB1 but not CB2 antagonists [260]. Local application of WIN 55,212-2 and a potent CB1 agonist, arachidonylcyclopropylamide, in the ventrolateral periaqueductal gray reduced dural-evoked Aδ-fiber neuronal activity in the Vc [261].

Systemic administration of cannabinoids was also effective in attenuating inflammatory and neuropathic pain in the orofacial region. The synthetic compound WIN 55,212-2, upon systemic administration, dose-dependently attenuated mechanical allodynia and thermal hyperalgesia in an NOP model induced by CCI of the infraorbital nerve [84]. Another synthetic cannabinoid, HU210, also attenuated mechanical allodynia and thermal hyperalgesia in that model. CB1 receptor but not CB2 or TRPV1 receptor antagonists inhibited the anti-nociceptive action of WIN 55,212-2, suggesting CB1 receptor-mediated action of this compound in that model [84]. The anti-nociceptive effects of WIN 55,212-2 were also observed in inflammatory pain models induced by injection of formalin into the temporomandibular joint and orofacial regions [262].

**Figure 1:**
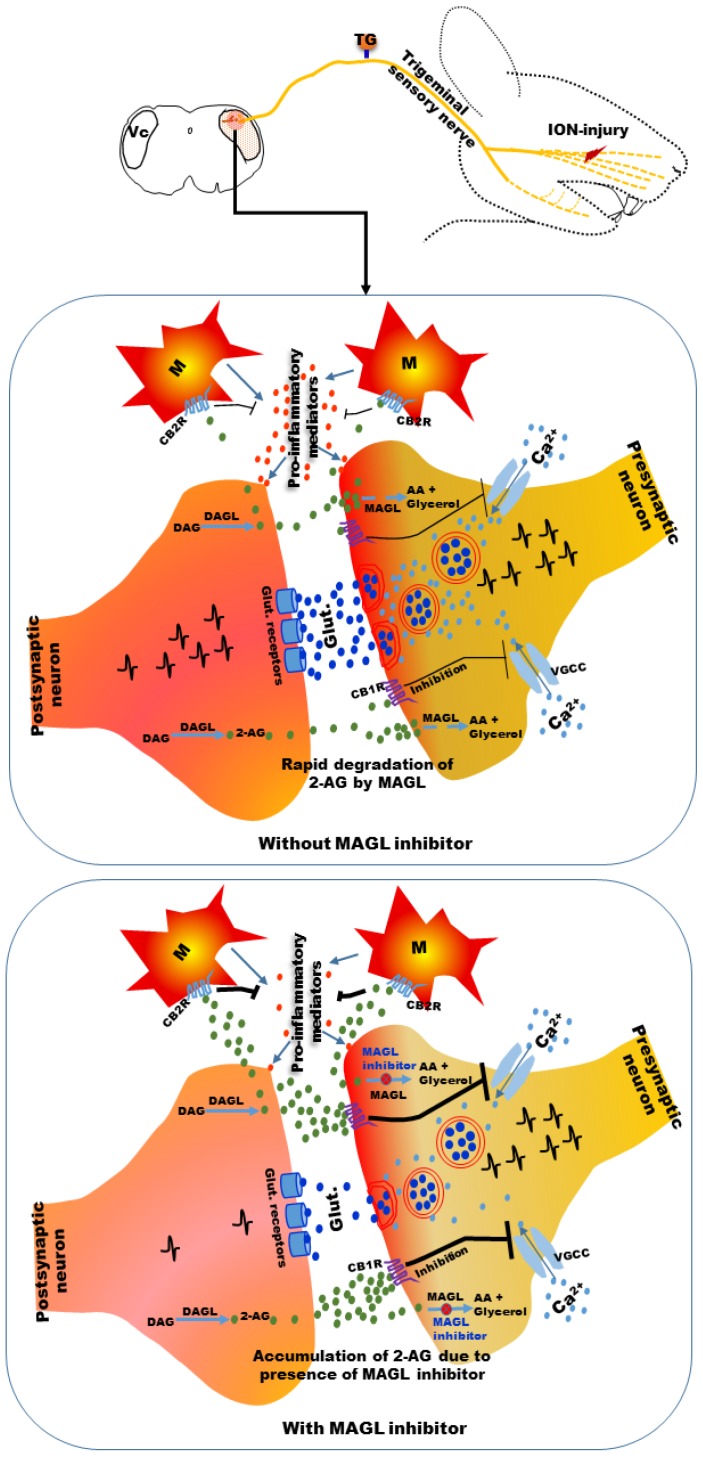
A possible mechanism of attenuation of neuropathic pain following infra-orbital nerve injury. 2-AG production in the brainstem trigeminal nucleus caudalis (Vc) may be increased by postsynaptic neurons because of exaggerated presynaptic neuronal activity under neuropathic pain conditions and rapidly degraded by MAGL. Prevention of the rapid degradation of 2-AG by JZL184 (an MAGL inhibitor) may increase its localized accumulation. Localized accumulation of 2-AG can act on CB1 receptors present in the presynaptic neurons and CB2 receptors present in microglia. Activation of CB1 receptors can inhibit voltage-gated Ca^2+^ channels (VGCCs), resulting in reduction of release of excitatory neurotransmitters from presynaptic neurons. Activation of CB2 receptors in microglia can inhibit the release of pro-inflammatory mediators. Reduction of release of excitatory neurotransmitters and pro-inflammatory mediators by accumulated 2-AG may cause attenuation of neuropathic pain symptoms. 2-AG: 2-arachydonoylglycerol; CB1R: cannabinoid 1 receptor; CB2R: cannabinoid 2 receptor; DAG: diacylglycerol; DAGL: diacylglycerol lipase; AA: arachidonic acid; MAGL: monoacylglycerol lipase; Glut.: glutamate; M: microglia.

Recently, in a study, we observed that a selective MAGL inhibitor, JZL184, reduced NOP induced by injury to a branch of the trigeminal nerve in mice [258]. Trigeminal nerve injury was observed to induce neuropathic pain symptoms in the orofacial area [258,263,264,265]. Systemic administration of the compound attenuated mechanical allodynia 2 h after administration [258]. We also observed that MAGL immunoreactive neurons were increased in the Vc and upper cervical spinal cord (C1-C2) under the neuropathic pain condition, and this effect was reduced after administration of JZL184 [258]. These observations suggest that 2-AG may be increased in the Vc and C1-C2 areas under neuropathic pain conditions (Figure 1) and rapidly degraded by MAGL, as indicated by the increased MAGL immunoreactivity [258].

2-AG can be released from postsynaptic neurons by exaggerated presynaptic neuronal activity because of nerve injury [21,70,258] (Figure 1), which can be considered as the body’s autoprotective/defense mechanism for pain control [21,61,68,69]. Exaggerated neuronal activity in the Vc and C1-C2 areas was evident in previous studies under NOP conditions following trigeminal nerve injuries [179]. The released 2-AG can act on CB1 receptors expressed on presynaptic neurons [266,267]. CB1 receptor activation may inhibit the VGCCs that are responsible for calcium entry into the presynaptic neurons, resulting in the release of neurotransmitters (e.g., glutamate) into the synaptic cleft [266,267] and thus can reduce neurotransmitter release. 2-AG can also act on cannabinoid receptors expressed on microglial cells [176,177,178,268,269]. Various studies have reported the presence of cannabinoid receptors in microglial cells [176,177,178,268,269], which predominantly express CB2 receptors [176,177,178,268,269]. Along with neurons, microglia may contribute to the synthesis and degradation of endocannabinoids [176,178,270]. Upregulated microglial cells in the brainstem trigeminal nuclei have been observed under NOP conditions to release pro-inflammatory mediators and contribute to the development of sensitization of brainstem neurons [113,179,263,264]. Activation of cannabinoid receptors in microglial cells by 2-AG may reduce the release of pro-inflammatory mediators. However, rapid degradation of 2-AG by MAGL may reduce these autoprotective mechanisms against pain control (reduction of presynaptic neurotransmitter release and reduction of pro-inflammatory mediator release from microglia by activation of cannabinoid receptors). Preventing the rapid degradation of 2-AG via MAGL inhibitor use may increase the localized accumulation of 2-AG in the Vc and C1-C2 areas. Localized accumulation of 2-AG acts on cannabinoid receptors, resulting in greater inhibition of VGCCs and glial cells, leading to reduction of excitatory neurotransmitter and pro-inflammatory mediator release and ultimately causing the attenuation of neuropathic pain symptoms (Figure 1) [258]. Additionally, prevention of the degradation of 2-AG to arachidonic acid and glycerol by MAGL inhibitors can reduce the amount of arachidonic acid, which is a precursor of inflammatory mediators (prostaglandins) [271]. Thus, reduction of arachidonic acid by MAGL inhibitors may contribute to attenuation of neuronal sensitization by inflammatory mediators.

The localized increase of endocannabinoids at the site of nerve injury may reduce the development of sensitization of peripheral nerves under neuropathic conditions. Studies have reported that nerve injury leads to activation of inflammatory cells at the site of nerve injury as well as in the TG under NOP conditions [113,179]. Inflammatory mediators released from these inflammatory cells contribute to the development of peripheral nerve sensitization [113,179]. A localized increase of endocannabinoids caused by applying degrading enzyme inhibitors can suppress the action of these inflammatory cells and thereby contribute to attenuation of neuropathic pain symptoms. The possible interaction of endocannabinoids with inflammatory and glial cells described above has been evidenced in neuropathic and inflammatory pain models of areas outside the orofacial regions [272,273,274]. 

The above studies in the orofacial regions suggested the potential of using cannabinoids and endocannabinoids for the treatment of NOP. However, few studies have been conducted, and recently developed peripherally restricted cannabinoid receptor agonists and endocannabinoid degrading enzyme inhibitors have not been extensively tested yet in models of NOP. Peripherally restricted CB1 receptor agonists, selective CB2 receptor agonists and some recently developed FAAH and MAGL inhibitors have been shown to have limited cannabimimetic side effects. These agents or newly designed agents should be tested in NOP models. Additionally, more randomized placebo-controlled trials are needed to determine the clinical utility of these agents in patients with NOP.

## 6. Clinical Perspective

Although an enormous body of preclinical research has provided convincing evidence of the anti-nociceptive effects of peripherally restricted CB1 receptor agonists, selective CB2 receptor agonists and endocannabinoid degrading enzyme inhibitors, clinical translation of these compounds for pain relief has remained elusive. Some FAAH inhibitors and selective CB1 and CB2 agonists have been tested in clinical studies but failed to produce sufficient anti-nociceptive effects, although some compounds were well tolerated and showed minimal or no adverse events [275,276,277]. A potent and selective FAAH inhibitor, PF04457845, did not produce better anti-nociceptive effects than the placebo when investigated in a randomized placebo controlled clinical trial in patients with osteoarthritis [275]. However, this compound was well tolerated, and there was no evidence of cannabinoid-type adverse events [275]. Another FAAH inhibitor, ASP8477, was used in a clinical trial of painful diabetic neuropathy, but it did not produce sufficient anti-nociceptive effects [278]. ASP3652 was investigated in patients with chronic prostatitis/chronic pelvic pain syndrome but failed to produce better anti-nociceptive effects than the placebo, although the compound was well tolerated by patients [276]. A clinical trial investigating an FAAH inhibitor, BIA10-2474, had devastating results; it produced mild to severe neurological symptoms and lead to the death of one volunteer [279,280]. The exact cause of this devastating event was unknown, but later it was observed that BIA10-2474 binds to a number of other serine hydrolases and inhibits several lipases that are not targeted by another FAAH inhibitor, PF04457845, which might cause substantial alterations in lipid networks in brain [281]. The selective CB1 agonist AZD1940 failed to reduce forearm capsaicin injection-induced pain and hyperalgesia in healthy volunteers and produced mild to moderate gastrointestinal and CNS side effects [95]. In another study, this compound failed to attenuate surgical tooth extraction-induced post-operative acute pain and produced CNS side effects [94]. The selective CB2 agonist GW842166 also failed to attenuate acute postsurgical pain in patients after third molar extraction, but it was well tolerated [277].

Failure of these compounds to produce sufficient anti-nociceptive effects in human trials suggests species differences in the action of these compounds; however, more research is necessary, and more new compounds need to be developed. Additional clinical trials focusing on neuropathic pain patients are necessary because the pathophysiology of neuropathic pain conditions is different from that of acute pain conditions. However, before clinical trials are conducted, extensive preclinical studies are required to understand the specific targets and side effects of these compounds. Although FAAH and MAGL are the main degrading enzymes of AEA and 2-AG, respectively, they can also degrade other lipids [158,164,204,281,282,283,284,285,286,287]. Along with AEA, FAAH can also degrade other N-acylethanolamines (e.g., N-palmitoylethanolamine (PEA) and N-oleoylethanolamine (OEA)), N-acyl-amides (e.g., N-acyl-taurines, N-acylglycines) and primary amides (e.g., oleamide), which can activate non-cannabinoid receptors (e.g., TRP channels, PPARα) [204,282,283,284,285]. Studies have reported that treatment with FAAH inhibitors increases the levels of AEA as well as OEA and PEA [204,282]. MAGL can also degrade other 2-acylglycerols (e.g., 2-linoleoyl glycerol, 2-oleoyl glycerol, 2-palmitoyl glycerol), which can activate non-CB1/-CB2 receptors [158,288,289]. Therefore, manipulation of FAAH and MAGL may also affect other lipids and may exert undesirable effects [158]. This important point should also be taken in consideration when designing compounds and investigating their effectiveness and side effects.

Additionally, laboratory animal models that can mimic key clinical symptoms of neuropathic pain need to be used to investigate the efficacy of the compounds [290]. Most of the available preclinical studies have investigated the efficacy of compounds for attenuation of allodynia and hyperalgesia in neuropathic pain models. Although allodynia and hyperalgesia are important symptoms, spontaneous pain is another key clinical symptom and complaint of most patients with neuropathic pain, which needs to be included in investigations [137,291]. Additionally, there are other important symptoms of neuropathic pain such as sleep disturbances, emotional disturbances and cognitive impairment [290]. Furthermore, neuropathic pain has been observed to develop more often in older individuals rather than in young people [292,293]. These factors need to be taken into consideration when establishing neuropathic pain models in animals.

Although the synthetic cannabinoid receptor agonists and endocannabinoid degrading enzyme inhibitors investigated in the above clinical trials failed to produce desired anti-nociceptive effects, several plant-based cannabinoids demonstrated some degree of analgesic effect on neuropathic pain. An oromucosal spray containing an equal mixture of THC (psychoactive component of *Cannabis*) and CBD (non-psychoactive component of *Cannabis*), nabiximols/Sativex, demonstrated significant anti-nociceptive effects in patients with peripheral neuropathic pain [294]. This drug has been approved in Canada and European countries. In a double-blind, randomized, placebo-controlled, parallel-group study, this drug was tested in patients with multiple sclerosis-induced central neuropathic pain. The patients were treated with the drug for 14 weeks. At 10 weeks, this drug significantly reduced multiple sclerosis-induced central neuropathic pain compared with the placebo; however, after 14 weeks, there was no significant difference between the placebo and the drug group [295]. In a pilot study, this drug showed no statistically significant anti-nociceptive effects when compared with a placebo in chemotherapy-induced neuropathic pain patients, although 5 of 16 patients reported some degree of pain reduction [296]. The combination of THC and CBD may be beneficial to reduce the cannabimimetic side effects of THC because CBD has been reported to reduce many of the psychotropic side effects of THC [27,297,298,299].

An oral formulation of THC (ECP002A) significantly reduced pain in patients with progressive multiple sclerosis [300]. A synthetic THC compound named Nabilone (approved by the Food and Drug Administration in the United States) was effective in reducing refractory diabetic peripheral neuropathic pain [301]. It also improved the sleep quality and overall status of patients [301]. Nabilone was also effective in improving sleep in patients with fibromyalgia (a disorder characterized by widespread musculoskeletal pain), although no effect on pain was observed [302]. Another clinical trial reported that this drug reduced medication overuse-induced headache [303]. It was more effective than an NSAID (ibuprofen) in reducing pain intensity and improved the quality of life [303]. The drug appeared to be safe and well-tolerated [303] in that study. The combination of this drug and gabapentin (an anticonvulsant) showed a better anti-nociceptive effect than the placebo or gabapentin alone in multiple sclerosis patients [304]. This THC derivative was given to patients taking opioids for chronic pain and found to produce an additive effect on pain relief [305].

Smoked cannabis was also observed to attenuate neuropathic pain [306,307,308]. A clinical trial involving patients with central and peripheral neuropathic pain reported that vaporized cannabis at low and medium doses attenuated neuropathic pain [306]. In another study, smoked cannabis produced significant anti-spasticity and anti-nociceptive effects in multiple sclerosis patients [307]. Smoked cannabis produced a dose-dependent, anti-nociceptive effect in patients with painful diabetic peripheral neuropathy [308].

Along with an anti-nociceptive effect, plant-based cannabinoid and smoked cannabis therapy showed cannabimimetic side effects, including cognitive impairment, euphoria, drowsiness or fatigue, dizziness, dry mouth, nausea, addiction, and motor impairment [22,57,309,310]. Therefore, a continuing need exists to identify therapeutics for neuropathic pain control with minimal or no side effects.

## 7. Conclusions

A large number of preclinical studies have provided evidence that targeting CB2 receptors, peripherally restricted CB1 receptors and endocannabinoid degrading enzymes is a potentially effective strategy to attenuate neuropathic pain symptoms with limited side effects. In particular, enhancing the action of endocannabinoids at the sites of the pain pathway using endocannabinoid degradative enzyme inhibitors is an attractive strategy for the treatment of neuropathic pain. The combination of endocannabinoid degradative enzyme inhibitors with conventional analgesics that have been used for the treatment of neuropathic pain (e.g., opioids) is also a promising strategy to produce synergistic anti-nociceptive effects and to reduce side effects. Studies examining neuropathic pain in the orofacial regions are scarce; therefore, more basic and clinical studies involving NOP are necessary to understand the efficacy and safety of these alternative strategies for neuropathic pain treatment.

## Figures and Tables

**Table 1:** Targeting peripherally localized cannabinoid 1 (CB1) receptors in preclinical animal models of neuropathic pain.

Compounds (Chemical Name)	Neuropathic Pain Models	Route of Administration	Anti-Nociceptive Effects	Antagonized by /Effectiveness in CB1/CB2 Knockout Mice	Cannabimimetic Side Effects	Ref.
ACEA [(5Z,8Z,11Z,14Z)-N-(2-Chloroethyl)-5,8,11,14-icosatetraen amide]	Spinal nerve ligation injury in rats	Intra-spinal	Attenuation of mechanically-evoked responses of spinal neurons	CB1-selective antagonist	Not tested	[87]
	Cisplatin-induced neuropathy in rats	Systemic/intra-plantar	Attenuation of mechanical allodynia	CB1-selective antagonist	Did not elicit psychoactive effect at the dose used	[88]
CRA13 [Naphthalen-1-yl-(4-pentyloxynaphthalen-1-yl) methanone]	Partial sciatic nerve ligation injury in rats	Oral	Attenuation of mechanical hyperalgesia	CB1-selective antagonist	Did not elicit cardiovascular and central nervous system (CNS) side effects at low but anti-nociceptive dose	[89]
AZD1940 [N-{1-[(4,4-Difluorocyclohexyl)methyl]-2-(2-methyl-2-propanyl)-1H-benzimidazol-5-yl} ethanesulfonamide]	Spinal nerve ligation injury in rats	Oral	Attenuation of mechanical allodynia	CB1-selective antagonist	Did not develop tolerance	[90]
AZ11713908 [N-{1-(Cyclohexylmethyl)-2-[(5-ethoxy-2-pyridinyl)methyl]-1H-benzimidazol-5-yl}-N-methyl-2-thiophenesulfonamide]	Spinal nerve ligation injury in rats	Systemic	Attenuation of mechanical allodynia	Not tested	Minimal CNS side effects	[91]
LBP1 [2-[4-({3-[7-Chloro-1-(tetrahydro-2H-pyran-4-ylmethyl)-1H-indol-3-yl]-1,2,4-oxadiazol-5-yl}methyl)-1-piperazinyl] acetamide]	Spinal nerve ligation injury in rats	Oral	Attenuation of thermal hyperalgesia and mechanical allodynia	CB1-selective antagonist	Did not elicit motor impairment	[92]
PrNMI [4-{2-[-(1E)-1[(4-propylnaphthalen-1-yl)methylidene]-1H-inden-3-yl]ethyl} morpholine]	Sciatic nerve entrapment injury in rats	Systemic/oral	Attenuation of mechanical allodynia	CB1-selective antagonist	Minimal CNS side effects	[64]

**Table 2:** Targeting the cannabinoid 2 (CB2) receptors in preclinical animal models of neuropathic pain.

Compounds [Chemical Name]	Neuropathic Pain Models	Route of Administration	Anti-Nociceptive Effects	Antagonized by/Effectiveness in CB1/CB2 Knockout Mice	Cannabimimetic Side Effects	Ref.
JWH015 [(2-Methyl-1-propyl-1H-indol-3-yl)(1-naphthyl) methanone]	Sciatic nerve chronic constriction injury (CCI) in mice	Intra-plantar	Attenuation of mechanical allodynia and thermal hyperalgesia	CB2-selective antagonist	Not tested	[131]
	Streptozotocin-induced diabetic neuropathy in mice	Intra-plantar	Attenuation of mechanical and thermal allodynia and thermal hyperalgesia	CB2-selective antagonist	Not tested	[132]
AM1241 [(2-Iodo-5-nitrophenyl) {1-[(1-methyl-2-piperidinyl)methyl]-1H-indol-3-yl} methanone]	Spinal nerve ligation injury in rats and mice	Systemic	Attenuation of mechanical allodynia and thermal hyperalgesia	CB2-selective antagonist, effective in CB1 knockout mice	Not tested	[101]
	Spinal nerve ligation injury in rats	Systemic/intra-spinal/intra-dorsal root ganglia (DRG)	Attenuation of mechanical allodynia	CB2-selective antagonist	Not tested	[103,108]
	Vincristine- induced neuropathy in rats	Systemic	Attenuation of mechanical allodynia	CB2-selective antagonist	Did not elicit catalepsy	[133]
	Paclitaxel- induced neuropathy in rats	Systemic	Attenuation of mechanical allodynia	CB2-selective antagonist	Not tested	[134]
	Streptozotocin- induced diabetic neuropathy in rats	Systemic	Attenuation of mechanical allodynia	Not tested	Not tested	[135,136]
	Spared nerve injury of sciatic nerve in rats	Systemic self-administration by animal	Attenuation of mechanical allodynia	Not tested	Did not elicit motor ataxia	[137]
	Sciatic nerve CCI in rats	Intra-spinal	Attenuation of mechanical allodynia	Not tested	Not tested	[138,139]
GW405833/L768242 [(2,3-Dichlorophenyl){5-methoxy-2-methyl-3-[2-(4-morpholinyl)ethyl]-1H-indol-1-yl} methanone]	Partial sciatic nerve ligation injury in rats	Systemic	Attenuation of mechanical allodynia	Not tested	Did not elicit sedation and catalepsy at low but effective dose	[127]
	Partial sciatic nerve ligation injury in mice	Systemic	Attenuation of mechanical allodynia	Not tested	High dose produced motor deficits	[129]
	Spinal nerve ligation injury in rats	Systemic	Attenuation of mechanical allodynia	CB2-selective antagonist	Not tested	[103]
	Modified spinal nerve ligation injury	Systemic (chronic)	Attenuation of mechanical allodynia	Not tested	Did not observe any motor deficits and catalepsy, although not formally measured	[126]
	Sciatic nerve CCI in rats	Systemic	Attenuation of mechanical allodynia and depression-like behavior	Not tested	Did not elicit motor impairment	[140]
MDA7 [1-[(3-benzyl-3-methyl-2,3-dihydro-1-benzofuran-6-yl)carbonyl] piperidine]	Paclitaxel- induced neuropathy in rats	Systemic	Attenuation of mechanical allodynia	CB2-selective antagonist	Did not affect locomotor activity	[141]
	Spinal nerve ligation injury in rats	Systemic	Attenuation of mechanical allodynia	CB2-selective antagonist	Did not affect locomotor activity	[141]
	Paclitaxel- induced neuropathy in rats and mice	Systemic (chronic)	Prevented the development of mechanical allodynia	CB2-selective antagonist, absent in CB2 knockout mice	Not tested	[117]
MDA19 [*N′*-[(3*Z*)-1-(1-hexyl)-2-oxo-1,2-dihydro -3*H*-indol-3-ylidene]benzo hydrazide]	Spinal nerve ligation injury in rats	Systemic	Attenuation of mechanical allodynia	CB2-selective antagonist	Did not affect locomotor activity	[142]
	Paclitaxel- induced neuropathy in rats and mice	Systemic	Attenuation of mechanical allodynia	Ineffective in CB2 knockout mice	Did not affect locomotor activity	[142]
NES400 [1-(2,4-dichlorophenyl)-6-methyl-N-cyclohexylamine-1,4-dihydroindeno[1,2-c]pyrazole-3-carboxamide]	Spared nerve injury in mice	Systemic (chronic)	Attenuation of mechanical allodynia and thermal hyperalgesia	Not tested	Not tested	[143]
JWH133 [(6aR,10aR)-6,6,9-Trimethyl-3-(2-methyl-2-pentanyl)-6a,7,10,10a-tetrahydro-6H -benzo[c] chromene]	Spinal nerve ligation injury in rats	Intra-spinal	Attenuation of mechanically evoked responses of spinal neurons	CB2-selective antagonist	Not tested	[87]
		Intra-plantar	Attenuation of mechanical stimuli-induced responses of WDR neurons in spinal cord	CB2-selective antagonist	Not tested	[121]
	Partial sciatic nerve ligation injury in mice	Intra-spinal	Attenuation of mechanical allodynia	Ineffective in CB2 receptor knockout mice	Not tested	[144]
		Subcutaneous	Attenuation of mechanical allodynia	CB2-selective antagonist	Not tested	[106]
	Cisplatin- induced neuropathy in rats	Systemic/intra-plantar	Attenuation of mechanical allodynia	CB2-selective antagonist	Did not elicit psychoactive effect at the dose used	[88]
AM1710 [1-Hydroxy-9-methoxy-3-(2-methyl-2-octanyl) -6H-benzo[c] chromen-6-one]	Cisplatin and paclitaxel-induced neuropathy in rats	Systemic	Attenuation of mechanical and cold allodynia	CB2-selective antagonist	Not tested	[145]
	Paclitaxel- induced neuropathy in rats	Subcutaneous (chronic)	Prevented the development of mechanical and cold allodynia	CB2-selective antagonist	Not tested	[146]
	Paclitaxel- induced neuropathy in mice	Systemic (acute and chronic)/intra-spinal	Attenuation of mechanical and cold allodynia	CB2-selective antagonist/ineffective in CB2 knockout mice	Did not elicit motor dysfunction and hypothermia	[147]
AM1714 [1,9-Dihydroxy-3-(2-methyl -2-octanyl)-6H-benzo[c] chromen-6-one]	Paclitaxel- induced neuropathy in rats	Systemic	Attenuation of mechanical allodynia	CB2-selective antagonist	Not tested	[134]
L759,656 [(6aR,10aR)-1-Methoxy-6,6-dimethyl-9-methylene-3-(2-methyl-2-octanyl)-6a,7,8,9,10,10a-hexahydro-6H-benzo[c] chromene]	Streptozotocin-induced diabetic neuropathy in mice	Intra-spinal	Attenuation of heat hyperalgesia	CB2-selective antagonist	Not tested	[148]
BCP [(1R,9S)-4,11,11-Trimethyl-8-methylenebicyclo [7.2.0] undec-4-ene]	Partial sciatic nerve ligation injury in mice	Oral (chronic)	Attenuation of mechanical allodynia and thermal hyperalgesia	Ineffective in CB2 knockout mice	Did not elicit motor disturbance, hypothermia or catalepsy	[106]
	Paclitaxel-induced neuropathy in mice	Oral (during and after paclitaxel treatment)	Attenuation of mechanical allodynia and prevention of development of mechanical allodynia	CB2 receptor antagonist	Not tested	[108]
LY2828360 [8-(2-Chlorophenyl)-2-methyl-6-(4-methyl-1-piperazinyl)-9-(tetrahydro-2H-pyran-4-yl)-9H -purine]	Paclitaxel- induced neuropathy in mice	Systemic (acute and chronic)	Attenuation of mechanical and cold allodynia	Not tested	Did not produce tolerance on chronic administration	[149]

**Table 3:** Targeting of FAAH for the treatment of neuropathic pain in preclinical animal models of neuropathic pain.

Compounds [Chemical Name]	Neuropathic Pain Models	Route of Administration	Anti-Nociceptive Effects	Antagonized by/Effectiveness in CB1/CB2/FAAH Knockout Mice	Cannabimimetic Side Effects	Ref.
OL135 [1-oxo-1-[5-(2-pyridyl)-2-yl]-7-phenyl heptane]	Sciatic nerve CCI in mice	Systemic	Attenuation of mechanical allodynia and acetone-induced cold allodynia	CB1 and CB2 receptor antagonists, not affected by the TRPV1 receptor antagonist	Not tested	[199]
		Oral	Attenuation of mechanical allodynia	Not tested	Not tested	[200]
URB597 [cyclohexylcarbamic acid 3′-carbamoylbiphenyl-3-yl ester]	Sciatic nerve CCI in mice	Systemic	Attenuation of mechanical allodynia and acetone-induced cold allodynia	CB1 and CB2 receptor antagonists, ineffective in fatty acid amide hydrolase (FAAH) knockout mice	Not tested	[199]
				Not tested	Did not elicit catalepsy and motor impairment but slight sedation observed	[201]
		Oral (chronic)	Attenuation of mechanical allodynia and thermal hyperalgesia	CB1 receptor antagonists	Not tested	[202]
		Oral	Attenuation of mechanical allodynia	Not tested	Not tested	[200]
	Sciatic nerve CCI in rats	Intra-spinal	Attenuation of mechanical allodynia and thermal hyperalgesia	CB1 or CB1/transient receptor potential vanilloid 1 (TRPV1) antagonists depending on the doses	Not tested	[203]
			Attenuation of mechanical allodynia, thermal hyperalgesia and cold allodynia	High dose antagonized by a TRPV1 antagonist	Not tested	[204]
	Partial sciatic nerve ligation injury in mice	Intra-plantar injection	Attenuation of mechanical allodynia and thermal hyperalgesia	CB1 receptor antagonists, ineffective in CB1 but not CB2 knockout mice	Not tested	[205]
	Partial sciatic nerve ligation injury in rats	Intra-plantar injection	Attenuation of mechanical allodynia and thermal hyperalgesia	CB1 and CB2 receptor antagonists	Not tested	[206]
		Intra- pre-limbic/infra-limbic cortex microinjection	Attenuation of mechanical allodynia	Not tested	Not tested	[207,208]
	Cisplatin- induced neuropathy in rats	Systemic	Attenuation of mechanical and cold allodynia	CB1, CB2, and TRPV1 receptor antagonists	Not tested	[183]
	Paclitaxel- induced neuropathy in mice	Systemic	Attenuation of mechanical and cold allodynia	Not tested	Not tested	[209]
	Paclitaxel- induced neuropathy in mice	Systemic (chronic)	Prevented the development of mechanical and cold allodynia and attenuated established allodynia	Not tested	No physical dependence or tolerance	[210]
	Streptozotocin-induced diabetic neuropathy in rats	Systemic	Attenuation of mechanical allodynia	Not tested	Not tested	[211]
ST4070 [1-biphenyl-4-ylethenyl piperidine-1-carboxylate]	Sciatic nerve CCI in mice	Oral	Attenuation of mechanical allodynia	CB1, CB2, and PPARα antagonists	Not tested	[200]
	Vincristine- induced neuropathy in mice	Oral	Attenuation of mechanical allodynia	Not tested	Not tested	[200]
	Streptozotocin-induced diabetic neuropathy in mice	Oral	Attenuation of mechanical allodynia	Not tested	Not tested	[200]
PF3845 [N-(3-Pyridinyl)-4-(3-{[5-(trifluoromethyl)-2-pyridinyl]oxy}benzyl)-1-piperidine carboxamide]	Sciatic nerve CCI in mice	Systemic	Attenuation of mechanical allodynia	Not tested	Did not elicit motor impairment, hypothermia and catalepsy	[212]
				Not tested	Not tested	[213]
			Attenuation of mechanical and cold allodynia	Not tested	Chronic administration did not induce development of tolerance	[214]
				Ineffective in CB1 and CB2 knockout mice	Not tested	[215]
	Sciatic nerve CCI in rat	Intra-spinal	Attenuation of mechanical allodynia and thermal hyperalgesia	Not tested	Not tested	[207]
URB937 [3′-Carbamoyl-6-hydroxy-3-biphenylyl cyclohexyl carbamate]	Sciatic nerve CCI in mice	Systemic	Attenuation of mechanical allodynia and thermal hyperalgesia	CB1 receptor antagonists	Did not alter spontaneous locomotor activity and daily food intake or feeding pattern	[216]
		Oral	Attenuation of mechanical allodynia, hyperalgesia and thermal hyperalgesia	Not tested	No gastric ulcerogenic effect	[217]
	Cisplatin- induced neuropathy in rats	Systemic	Attenuation of mechanical and cold allodynia	CB1, CB2, and TRPV1 receptor antagonists	Not tested	[183]
	Paclitaxel- induced neuropathy in mice	Systemic (chronic)	Prevented the development of mechanical and cold allodynia and attenuated established allodynia	Not tested	No physical dependence or tolerance	[210]

**Table 4:** Targeting MAGL for the treatment of neuropathic pain in preclinical animal models of neuropathic pain.

Compounds [Chemical Name]	Neuropathic Pain Models	Route of Administration	Anti-Nociceptive Effects	Antagonized by/Effectiveness in CB1/CB2 Knockout Mice	Cannabimimetic Side Effects	Ref.
URB602 [1,1′-biphenyl]-3-yl- carbamic acid, cyclohexyl ester]	Partial sciatic nerve ligation in rats	Intra-plantar	Attenuation of mechanical allodynia and thermal hyperalgesia	CB1 and CB2 receptor antagonists	Not tested	[206]
	Partial sciatic nerve ligation in mice	Intra-plantar	Attenuation of mechanical allodynia and thermal hyperalgesia	CB1 and CB2 receptor antagonists, the effect was altered in both CB1 and CB2 knockout mice	Not tested	[205]
JZl184 [4-nitrophenyl4-(dibenzo[d][1,3]dioxol-5-yl (hydroxy) methyl) piperidine-1- carboxylate]	Sciatic nerve CCI injury in mice	Systemic	Attenuation of mechanical and cold allodynia	CB1 antagonist	Produced hypo-motility	[199]
				Ineffective in CB1 but not CB2 knockout mice	Not tested	[215]
				Not tested	Chronic administration induced tolerance, impairment of synaptic plasticity and downregulation and desensitization of CB1 receptors	[214]
				Not tested	Not tested	[223]
				Not tested	Not tested	[224]
				Not tested	Not tested	[225]
				Not tested	Catalepsy, motor impairment and sedation	[201]
			Attenuation of mechanical and thermal hyperalgesia	Mechanical allodynia antagonized by both CB1 and CB2 receptor antagonists and thermal hyperalgesia antagonized by CB1 receptor antagonist	Produced hypo-motility	[226]
			Attenuation of mechanical allodynia	Not tested	High dose but not low dose produced impairment of locomotor activity, hypothermia and catalepsy	[212]
	Paclitaxel- induced neuropathy in mice	Systemic	Attenuation of mechanical allodynia	CB1 and CB2 receptor antagonists, ineffective in both CB1 and CB2 knockout mice	Not tested	[227]
			Attenuation of mechanical and cold allodynia	Not tested	Not tested	[209]
	Cisplatin- induced neuropathy in rats	Systemic	Attenuation of mechanical and cold allodynia	CB1 and CB2 receptor antagonists	Not tested	[183]
	Cisplatin- induced neuropathy in mice	Intra-planter (chronic)	Attenuation of mechanical hyperalgesia	CB1 but not CB2 receptor antagonists	Not tested	[228]
KML29 [1,1,1,3,3,3-hexafluoropropan-2-yl 4-(bis(benzo[*d*][1,3]dioxol-5-yl)(hydroxy)methyl)piperidine-1-carboxylate]	Sciatic nerve CCI in mice	Systemic	Attenuation of mechanical and cold allodynia	CB1 receptor antagonist	Did not elicit catalepsy, hypothermia and hypo-motility	[224]
			Attenuation of mechanical and cold allodynia	Not tested	Not tested	[225]
MJN110 [2,5-dioxopyrrolidin-1-yl 4-(bis(4-chlorophenyl) methyl)piperazine-1- carboxylate]	Sciatic nerve CCI injury in mice	Systemic	Attenuation of mechanical allodynia and thermal hyperalgesia	Mechanical allodynia antagonized by both CB1 and CB2 receptor antagonists and thermal hyperalgesia antagonized by CB1 receptor antagonist	Did not elicit hypo-motility, catalepsy and hypothermia	[226]
	Streptozotocin-induced diabetic neuropathy in rats	Systemic	Attenuation of mechanical allodynia	Not tested	Not tested	[211]
	Paclitaxel- induced neuropathy in mice	Systemic	Attenuation of mechanical allodynia	CB1 and CB2 receptor antagonists, ineffective in both CB1 and CB2 knockout mice	Not tested	[227]

**Table 5:** Research conducted using cannabinoid receptor agonists and endocannabinoid enzyme inhibitors in preclinical animal models of neuropathic orofacial pain (NOP).

Compounds	Neuropathic Pain Models	Route of Administration	Anti-Nociceptive Effects	Antagonized by	Cannabimimetic Side Effects	Ref.
WIN 55,212-2 (synthetic cannabinoid)	Infra-orbital nerve CCI in rats	Systemic	Attenuation of mechanical allodynia and thermal hyperalgesia	CB1 but not CB2 antagonist	Not tested	[84]
HU210 (synthetic cannabinoid)	Infra-orbital nerve CCI in rats	Systemic	Attenuation of mechanical allodynia and thermal hyperalgesia	Not tested	Not tested	[84]
AM1241 (selective CB2 agonist)	Nitroglycerin- induced migraine model in rats	Systemic	Attenuation of nocifensive behavior induced by formalin injection into paw	Not tested	Not tested	[256]
URB597 (FAAH inhibitor)	Nitroglycerin- induced migraine model in mice	Systemic	Attenuation of mechanical hyperalgesia	CB1 receptor antagonist	Not tested	[248]
PF3845 (FAAH inhibitor)	Nitroglycerin- induced migraine model in mice	Systemic	Attenuation of mechanical hyperalgesia	CB1 receptor antagonist	Not tested	[248]
URB937 (FAAH inhibitor)	Nitroglycerin- induced migraine model in rats	Systemic	Attenuation of nocifensive behavior induced by formalin injection into lip and paw	Not tested	Not tested	[257]
JZL184 (MAGL inhibitor)	Partial transection of infra-orbital nerve in mice	Systemic	Attenuation of mechanical allodynia	Not tested	Not tested	[258]

## References

[1] Murnion B.P. (2018). Neuropathic pain: Current definition and review of drug treatment. Aust. Prescr..

[2] Merskey H. (1994). Part III Pain Terms, A Current List with Definitions and Notes on usage. Classification Chronic Pain-Descriptions Chronic Pain Syndromes and Definitions of Pain Terms.

[3] Dueñas M., Ojeda B., Salazar A., Mico J.A., Failde I. (2016). A review of chronic pain impact on patients, their social environment and the health care system. J. Pain Res..

[4] McDermott A.M., Toelle T.R., Rowbotham D.J., Schaefer C.P., Dukes E.M. (2006). The burden of neuropathic pain: Results from a cross-sectional survey. Eur. J. Pain.

[5] Schaefer C., Mann R., Sadosky A., Daniel S., Parsons B., Nieshoff E., Tuchman M., Nalamachu S., Anschel A., Stacey B.R. (2014). Burden of illness associated with peripheral and central neuropathic pain among adults seeking treatment in the united states: A patient-centered evaluation. Pain Med..

[6] Benoliel R., Sharav Y. (2010). Chronic orofacial pain. Curr. Pain Headache Rep..

[7] Sessle B.J. (2000). Acute and chronic craniofacial pain: Brainstem mechanisms of nociceptive transmission and neuroplasticity, and their clinical correlates. Crit. Rev. Oral Biol. Med..

[8] Maarbjerg S., Di Stefano G., Bendtsen L., Cruccu G. (2017). Trigeminal neuralgia—Diagnosis and treatment. Cephalalgia.

[9] Zakrzewska J.M. (2013). Differential diagnosis of facial pain and guidelines for management. Br. J. Anaesth..

[10] Macfarlane T.V., Blinkhorn A.S., Davies R.M., Ryan P., Worthington H.V., Macfarlane G.J. (2002). Orofacial pain: Just another chronic pain? Results from a population-based survey. Pain.

[11] Mueller D., Obermann M., Yoon M.S., Poitz F., Hansen N., Slomke M.A., Dommes P., Gizewski E., Diener H.C., Katsarava Z. (2011). Prevalence of trigeminal neuralgia and persistent idiopathic facial pain: A population-based study. Cephalalgia.

[12] Koopman J.S.H.A., Dieleman J.P., Huygen F.J., de Mos M., Martin C.G.M., Sturkenboom M.C.J.M. (2009). Incidence of facial pain in the general population. Pain.

[13] Finnerup N.B., Attal N., Haroutounian S., McNicol E., Baron R., Dworkin R.H., Gilron I., Haanpää M., Hansson P., Jensen T.S. (2015). Pharmacotherapy for neuropathic pain in adults: A systematic review and meta-analysis. Lancet Neurol..

[14] Fornasari D. (2017). Pharmacotherapy for Neuropathic Pain: A Review. Pain Ther..

[15] Torrance N., Ferguson J.A., Afolabi E., Bennett M.I., Serpell M.G., Dunn K.M., Smith B.H. (2013). Neuropathic pain in the community: More under-treated than refractory?. Pain.

[16] Guirguis-Blake J., Kelly C. (2007). Are opioids effective in the treatment of neuropathic pain?. Am. Fam. Physician.

[17] Thomas D.A., Frascella J., Hall T., Smith W., Compton W., Koroshetz W., Briggs J., Grady P., Somerman M., Volkow N. (2015). Reflections on the role of opioids in the treatment of chronic pain: A shared solution for prescription opioid abuse and pain. J. Intern. Med..

[18] Russo E.B. (2008). Cannabinoids in the management of difficult to treat pain. Ther. Clin. Risk Manag..

[19] Lau B.K., Vaughan C.W. (2014). Targeting the endogenous cannabinoid system to treat neuropathic pain. Front. Pharmacol..

[20] Donvito G., Nass S.R., Wilkerson J.L., Curry Z.A., Schurman L.D., Kinsey S.G., Lichtman A.H. (2018). The Endogenous Cannabinoid System: A Budding Source of Targets for Treating Inflammatory and Neuropathic Pain. Neuropsychopharmacology.

[21] Jhaveri M.D., Richardson D., Chapman V. (2007). Endocannabinoid metabolism and uptake: Novel targets for neuropathic and inflammatory pain. Br. J. Pharmacol..

[22] Jensen B., Chen J., Furnish T., Wallace M. (2015). Medical Marijuana and Chronic Pain: A Review of Basic Science and Clinical Evidence. Curr. Pain Headache Rep..

[23] Rahn E.J., Hohmann A.G. (2009). Cannabinoids as Pharmacotherapies for Neuropathic Pain: From the Bench to the Bedside. Neurotherapeutics.

[24] Gaoni Y., Mechoulam R. (1964). Isolation, Structure, and Partial Synthesis of an Active Constituent of Hashish. J. Am. Chem. Soc..

[25] Mechoulam R., Shvo Y. (1963). Hashish-I. The structure of Cannabidiol. Tetrahedron.

[26] Radwan M.M., ElSohly M.A., Slade D., Ahmed S.A., Khan I.A., Ross S.A. (2009). Biologically active cannabinoids from high-potency Cannabis sativa. J. Nat. Prod..

[27] Schubart C.D., Sommer I.E.C., van Gastel W.A., Goetgebuer R.L., Kahn R.S., Boks M.P.M. (2011). Cannabis with high cannabidiol content is associated with fewer psychotic experiences. Schizophr. Res..

[28] Zuardi A.W. (2006). History of cannabis as a medicine: A review. Rev. Bras. Psiquiatr..

[29] Devane W.A., Hanuš L., Breuer A., Pertwee R.G., Stevenson L.A., Griffin G., Gibson D., Mandelbaum A., Etinger A., Mechoulam R. (1992). Isolation and structure of a brain constituent that binds to the cannabinoid receptor. Science.

[30] Lu H.C., MacKie K. (2016). An introduction to the endogenous cannabinoid system. Biol. Psychiatry.

[31] Liu J., Wang L., Harvey-White J., Osei-Hyiaman D., Razdan R., Gong Q., Chan A.C., Zhou Z., Huang B.X., Kim H.Y. (2006). A biosynthetic pathway for anandamide. Proc. Natl. Acad. Sci. USA.

[32] Murataeva N., Straiker A., MacKie K. (2014). Parsing the players: 2-arachidonoylglycerol synthesis and degradation in the CNS. Br. J. Pharmacol..

[33] Ueda N., Tsuboi K., Uyama T., Ohnishi T. (2011). Biosynthesis and degradation of the endocannabinoid 2-arachidonoylglycerol. BioFactors.

[34] Di Marzo V. (2006). Endocannabinoids: Synthesis and Degradation. Reviews of Physiology Biochemistry and Pharmacology.

[35] Ahn K., McKinney M.K., Cravatt B.F. (2008). Enzymatic pathways that regulate endocannabinoid signaling in the nervous system. Chem. Rev..

[36] Munro S., Thomas K.L., Abu-Shaar M. (1993). Molecular characterization of a peripheral receptor for cannabinoids. Nature.

[37] Matsuda L.A., Lolait S.J., Brownstein M.J., Young A.C., Bonner T.I. (1990). Structure of a cannabinoid receptor and functional expression of the cloned cDNA. Nature.

[38] Morales P., Reggio P.H. (2017). An Update on Non-CB 1, Non-CB 2 Cannabinoid Related G-Protein-Coupled Receptors. Cannabis Cannabinoid Res..

[39] O’Sullivan S.E. (2016). An update on PPAR activation by cannabinoids. Br. J. Pharmacol..

[40] Fenwick A.J., Fowler D.K., Wu S.W., Shaffer F.J., Lindberg J.E.M., Kinch D.C., Peters J.H. (2017). Direct anandamide activation of TRPV1 produces divergent calcium and current responses. Front. Mol. Neurosci..

[41] Brown S.P., Safo P.K., Regehr W.G. (2004). Endocannabinoids inhibit transmission at granule cell to Purkinje cell synapses by modulating three types of presynaptic calcium channels. J. Neurosci..

[42] Guo J., Ikeda S.R. (2004). Endocannabinoids Modulate N-Type Calcium Channels and G-Protein-Coupled Inwardly Rectifying Potassium Channels via CB1 Cannabinoid Receptors Heterologously Expressed in Mammalian Neurons. Mol. Pharmacol..

[43] Zou S., Kumar U. (2018). Cannabinoid receptors and the endocannabinoid system: Signaling and function in the central nervous system. Int. J. Mol. Sci..

[44] Deadwyler S.A., Hampson R.E., Mu J., Whyte A., Childers S. (1995). Cannabinoids modulate voltage sensitive potassium A-current in hippocampal neurons via a cAMP-dependent process. J. Pharmacol. Exp. Ther..

[45] Felder C.C., Joyce K.E., Briley E.M., Mansouri J., Mackie K., Blond O., Lai Y., Ma A.L., Mitchell R.L. (1995). Comparison of the pharmacology and signal transduction of the human cannabinoid CB1 and CB2 receptors. Mol. Pharmacol..

[46] Devane W.A., Dysarz F.A., Johnson M.R., Melvin L.S., Howlett A.C. (1988). Determination and characterization of a cannabinoid receptor in rat brain. Mol. Pharmacol..

[47] Galiègue S., Mary S., Marchand J., Dussossoy D., Carrière D., Carayon P., Bouaboula M., Shire D., LE Fur G., Casellas P. (1995). Expression of Central and Peripheral Cannabinoid Receptors in Human Immune Tissues and Leukocyte Subpopulations. Eur. J. Biochem..

[48] Hohmann A.G., Herkenham M. (1999). Localization of central cannabinoid CB1 receptor messenger RNA in neuronal subpopulations of rat dorsal root ganglia: A double-label in situ hybridization study. Neuroscience.

[49] Bridges D., Rice A.S.C., Egertová M., Elphick M.R., Winter J., Michael G.J. (2003). Localisation of cannabinoid receptor 1 in rat dorsal root ganglion using in situ hybridisation and immunohistochemistry. Neuroscience.

[50] Price T.J., Helesic G., Parghi D., Hargreaves K.M., Flores C.M. (2003). The neuronal distribution of cannabinoid receptor type 1 in the trigeminal ganglion of the rat. Neuroscience.

[51] Ahluwalia J., Urban L., Capogna M., Bevan S., Nagy I. (2000). Cannabinoid 1 receptors are expressed in nociceptive primary sensory neurons. Neuroscience.

[52] Agarwal N., Pacher P., Tegeder I., Amaya F., Constantin C.E., Brenner G.J., Rubino T., Michalski C.W., Marsicano G., Monory K. (2007). Cannabinoids mediate analgesia largely via peripheral type 1 cannabinoid receptors in nociceptors. Nat. Neurosci..

[53] Hill K.P., Palastro M.D., Johnson B., Ditre J.W. (2017). Cannabis and Pain: A Clinical Review. Cannabis Cannabinoid Res..

[54] Jagerovic N., Hernandez-Folgado L., Goya P., Jagerovic N., Hernandez-Folgado L., Martin M.I. (2003). Cannabinoids and Neuropathic Pain. Mini Rev. Med. Chem..

[55] Lynch M.E., Campbell F. (2011). Cannabinoids for treatment of chronic non-cancer pain; a systematic review of randomized trials. Br. J. Clin. Pharmacol..

[56] Pertwee R.G. (2001). Cannabinoid receptors and pain. Prog. Neurobiol..

[57] Volkow N.D., Baler R.D., Compton W.M., Weiss S.R.B. (2014). Adverse health effects of marijuana use. N. Engl. J. Med..

[58] Thomas H. (1996). A community survey of adverse effects of cannabis use. Drug Alcohol Depend..

[59] Kalant H. (2004). Adverse effects of cannabis on health: An update of the literature since 1996. Prog. Neuro Psychopharmacol. Biol. Psychiatry.

[60] Moreira F.A., Grieb M., Lutz B. (2009). Central side-effects of therapies based on CB1 cannabinoid receptor agonists and antagonists: Focus on anxiety and depression. Best Pract. Res. Clin. Endocrinol. Metab..

[61] Spigelman I. (2010). Therapeutic Targeting of Peripheral Cannabinoid Receptors in Inflammatory and Neuropathic Pain States. Translational Pain Research: From Mouse to Man.

[62] Kunos G., Osei-Hyiaman D., Bátkai S., Sharkey K.A., Makriyannis A. (2009). Should peripheral CB1 cannabinoid receptors be selectively targeted for therapeutic gain?. Trends Pharmacol. Sci..

[63] Banister S.D., Krishna Kumar K., Kumar V., Kobilka B.K., Malhotra S.V. (2019). Selective modulation of the cannabinoid type 1 (CB1) receptor as an emerging platform for the treatment of neuropathic pain. Medchemcomm.

[64] Seltzman H.H., Shiner C., Hirt E.E., Gilliam A.F., Thomas B.F., Maitra R., Snyder R., Black S.L., Patel P.R., Mulpuri Y. (2016). Peripherally Selective Cannabinoid 1 Receptor (CB1R) Agonists for the Treatment of Neuropathic Pain. J. Med. Chem..

[65] Whiteside G., Lee G., Valenzano K. (2007). The Role of the Cannabinoid CB2 Receptor in Pain Transmission and Therapeutic Potential of Small Molecule CB2 Receptor Agonists. Curr. Med. Chem..

[66] Guindon J., Hohmann A.G. (2008). Cannabinoid CB 2 receptors: A therapeutic target for the treatment of inflammatory and neuropathic pain. Br. J. Pharmacol..

[67] Guindon J., Hohmann A. (2012). The Endocannabinoid System and Pain. CNS Neurol. Disord. Drug Targets.

[68] Woodhams S.G., Sagar D.R., Burston J.J., Chapman V. (2015). The role of the endocannabinoid system in pain. Handb. Exp. Pharmacol..

[69] Pertwee R.G. (2012). Targeting the endocannabinoid system with cannabinoid receptor agonists: Pharmacological strategies and therapeutic possibilities. Philos. Trans. R. Soc. B Biol. Sci..

[70] Alger B.E., Kim J. (2011). Supply and demand for endocannabinoids. Trends Neurosci..

[71] Lichtman A.H., Cook S.A., Martin B.R. (1996). Investigation of brain sites mediating cannabinoid-induced antinociception in rats: Evidence supporting periaqueductal gray involvement. J. Pharmacol. Exp. Ther..

[72] Van Sickle M.D., Oland L.D., Ho W., Hillard C.J., Mackie K., Davison J.S., Sharkey K.A. (2001). Cannabinoids inhibit emesis through CB1 receptors in the brainstem of the ferret. Gastroenterology.

[73] Sañudo-Peña M.C., Strangman N.M., Mackie K., Walker J.M., Kang T. (1999). CB1 receptor localization in rat spinal cord and roots, dorsal root ganglion, and peripheral nerve. Acta Pharmacol. Sin..

[74] Binzen U., Greffrath W., Hennessy S., Bausen M., Saaler-Reinhardt S., Treede R.D. (2006). Co-expression of the voltage-gated potassium channel Kv1.4 with transient receptor potential channels (TRPV1 and TRPV2) and the cannabinoid receptor CB1 in rat dorsal root ganglion neurons. Neuroscience.

[75] Mostafeezur R.M., Zakir H.M., Takatsuji H., Yamada Y., Yamamura K., Kitagawa J. (2012). Cannabinoids Facilitate the Swallowing Reflex Elicited by the Superior Laryngeal Nerve Stimulation in Rats. PLoS ONE.

[76] Khasabova I.A., Simone D.A., Seybold V.S. (2002). Cannabinoids attenuate depolarization-dependent Ca2+ influx in intermediate-size primary afferent neurons of adult rats. Neuroscience.

[77] Mitrirattanakul S., Ramakul N., Guerrero A.V., Matsuka Y., Ono T., Iwase H., Mackie K., Faull K.F., Spigelman I. (2006). Site-specific increases in peripheral cannabinoid receptors and their endogenous ligands in a model of neuropathic pain. Pain.

[78] Ständer S., Schmelz M., Metze D., Luger T., Rukwied R. (2005). Distribution of cannabinoid receptor 1 (CB1) and 2 (CB2) on sensory nerve fibers and adnexal structures in human skin. J. Dermatol. Sci..

[79] Amaya F., Shimosato G., Kawasaki Y., Hashimoto S., Tanaka Y., Ji R.R., Tanaka M. (2006). Induction of CB1 cannabinoid receptor by inflammation in primary afferent neurons facilitates antihyperalgesic effect of peripheral CB1 agonist. Pain.

[80] Lim G., Sung B., Ji R.R., Mao J. (2003). Upregulation of spinal cannabinoid-1-receptors following nerve injury enhances the effects of Win 55,212-2 on neuropathic pain behaviors in rats. Pain.

[81] Siegling A., Hofmann H.A., Denzer D., Mauler F., De Vry J. (2001). Cannabinoid CB1 receptor upregulation in a rat model of chronic neuropathic pain. Eur. J. Pharmacol..

[82] Walczak J.S., Pichette V., Leblond F., Desbiens K., Beaulieu P. (2006). Characterization of chronic constriction of the saphenous nerve, a model of neuropathic pain in mice showing rapid molecular and electrophysiological changes. J. Neurosci. Res..

[83] Walczak J.S., Pichette V., Leblond F., Desbiens K., Beaulieu P. (2005). Behavioral, pharmacological and molecular characterization of the saphenous nerve partial ligation: A new model of neuropathic pain. Neuroscience.

[84] Liang Y.C., Huang C.C., Hsu K. (2007). Sen The synthetic cannabinoids attenuate allodynia and hyperalgesia in a rat model of trigeminal neuropathic pain. Neuropharmacology.

[85] Fox A., Kesingland A., Gentry C., McNair K., Patel S., Urban L., James I. (2001). The role of central and peripheral Cannabinoid1 receptors in the antihyperalgesic activity of cannabinoids in a model of neuropathic pain. Pain.

[86] Potenzieri C., Brink T.S., Pacharinsak C., Simone D.A. (2008). Cannabinoid modulation of cutaneous Aδ nociceptors during inflammation. J. Neurophysiol..

[87] Sagar D.R., Kelly S., Millns P.J., O’Shaughnessey C.T., Kendall D.A., Chapman V. (2005). Inhibitory effects of CB1 and CB2 receptor agonists on responses of DRG neurons and dorsal horn neurons in neuropathic rats. Eur. J. Neurosci..

[88] Vera G., Cabezos P.A., Martín M.I., Abalo R. (2013). Characterization of cannabinoid-induced relief of neuropathic pain in a rat model of cisplatin-induced neuropathy. Pharmacol. Biochem. Behav..

[89] Dziadulewicz E.K., Bevan S.J., Brain C.T., Coote P.R., Culshaw A.J., Davis A.J., Edwards L.J., Fisher A.J., Fox A.J., Gentry C. (2007). Naphthalen-1-yl-(4-pentyloxynaphthalen-1-yl)methanone: A potent, orally bioavailable human CB1/CB2 dual agonist with antihyperalgesic properties and restricted central nervous system penetration. J. Med. Chem..

[90] Groblewski T., Yu X.H., Lessard E., St-Onge S., Yang H., Panetta R., Cao C.Q., Swedberg M., Cebers G., Nyberg S. Pre-clinical pharmacological properties of novel peripherally-acting CB1-CB2 agonists. Proceedings of the 20th Annual Symposium Cannabinoids.

[91] Yu X.H., Cao C.Q., Martino G., Puma C., Morinville A., St-Onge S., Lessard É., Perkins M.N., Laird J.M.A. (2010). A peripherally restricted cannabinoid receptor agonist produces robust anti-nociceptive effects in rodent models of inflammatory and neuropathic pain. Pain.

[92] Adam J.M., Clark J.K., Davies K., Everett K., Fields R., Francis S., Jeremiah F., Kiyoi T., Maidment M., Morrison A. (2012). Low brain penetrant CB1 receptor agonists for the treatment of neuropathic pain. Bioorganic Med. Chem. Lett..

[93] Page D., Wei Z., Liu Z., Tremblay M., Desfosses H., Milburn C., Srivastava S., Yang H., Brown W., Walpole C. (2010). 5-Sulfonamide Benzimidazoles: A Class of Cannabinoid Receptors Agonists with Potent In Vivo Antinociception Activity. Lett. Drug Des. Discov..

[94] Kalliomäki J., Segerdahl M., Webster L., Reimfelt A., Huizar K., Annas P., Karlsten R., Quiding H. (2013). Evaluation of the analgesic efficacy of AZD1940, a novel cannabinoid agonist, on post-operative pain after lower third molar surgical removal. Scand. J. Pain.

[95] Kalliomäki J., Annas P., Huizar K., Clarke C., Zettergren A., Karlsten R., Segerdahl M. (2013). Evaluation of the analgesic efficacy and psychoactive effects of AZD1940, a novel peripherally acting cannabinoid agonist, in human capsaicin-induced pain and hyperalgesia. Clin. Exp. Pharmacol. Physiol..

[96] Lynn A.B., Herkenham M. (1994). Localization of cannabinoid receptors and nonsaturable high-density cannabinoid binding sites in peripheral tissues of the rat: Implications for receptor-mediated immune modulation by cannabinoids. J. Pharmacol. Exp. Ther..

[97] Griffin G., Fernando S.R., Ross R.A., McKay N.G., Ashford M.L.J., Shire D., Huffman J.W., Yu S., Lainton J.A.H., Pertwee R.G. (1997). Evidence for the presence of CB2-1ike cannabinoid receptors on peripheral nerve terminals. Eur. J. Pharmacol..

[98] Van Sickle M.D., Duncan M., Kingsley P.J., Mouihate A., Urbani P., Mackie K., Stella N., Makriyannis A., Piomelli D., Davison J.S. (2005). Neuroscience: Identification and functional characterization of brainstem cannabinoid CB2 receptors. Science.

[99] Wotherspoon G., Fox A., McIntyre P., Colley S., Bevan S., Winter J. (2005). Peripheral nerve injury induces cannabinoid receptor 2 protein expression in rat sensory neurons. Neuroscience.

[100] Gong J.P., Onaivi E.S., Ishiguro H., Liu Q.R., Tagliaferro P.A., Brusco A., Uhl G.R. (2006). Cannabinoid CB2 receptors: Immunohistochemical localization in rat brain. Brain Res..

[101] Ibrahim M.M., Deng H., Zvonok A., Cockayne D.A., Kwan J., Mata H.P., Vanderah T.W., Lai J., Porreca F., Makriyannis A. (2003). Activation of CB2 cannabinoid receptors by AM1241 inhibits experimental neuropathic pain: Pain inhibition by receptors not present in the CNS. Proc. Natl. Acad. Sci. USA.

[102] Stumpf A., Parthier D., Sammons R.P., Stempel A.V., Breustedt J., Rost B.R., Schmitz D. (2018). Cannabinoid type 2 receptors mediate a cell type-specific self-inhibition in cortical neurons. Neuropharmacology.

[103] Beltramo M., Bernardini N., Bertorelli R., Campanella M., Nicolussi E., Fredduzzi S., Reggiani A. (2006). CB2 receptor-mediated antihyperalgesia: Possible direct involvement of neural mechanisms. Eur. J. Neurosci..

[104] Hsieh G.C., Pai M., Chandran P., Hooker B.A., Zhu C.Z., Salyers A.K., Wensink E.J., Zhan C., Carroll W.A., Dart M.J. (2011). Central and peripheral sites of action for CB 2 receptor mediated analgesic activity in chronic inflammatory and neuropathic pain models in rats. Br. J. Pharmacol..

[105] Zhang J., Hoffert C., Vu H.K., Groblewski T., Ahmad S., O’Donnell D. (2003). Induction of CB2 receptor expression in the rat spinal cord of neuropathic but not inflammatory chronic pain models. Eur. J. Neurosci..

[106] Klauke A.L., Racz I., Pradier B., Markert A., Zimmer A.M., Gertsch J., Zimmer A. (2014). The cannabinoid CB2 receptor-selective phytocannabinoid beta-caryophyllene exerts analgesic effects in mouse models of inflammatory and neuropathic pain. Eur. Neuropsychopharmacol..

[107] Racz I., Nadal X., Alferink J., Baños J.E., Rehnelt J., Martín M., Pintado B., Gutierrez-Adan A., Sanguino E., Manzanares J. (2008). Crucial role of CB2 cannabinoid receptor in the regulation of central immune responses during neuropathic pain. J. Neurosci..

[108] Segat G.C., Manjavachi M.N., Matias D.O., Passos G.F., Freitas C.S., Costa R., Calixto J.B. (2017). Antiallodynic effect of β-caryophyllene on paclitaxel-induced peripheral neuropathy in mice. Neuropharmacology.

[109] Benito C., Kim W.K., Chavarría I., Hillard C.J., Mackie K., Tolón R.M., Williams K., Romero J. (2005). A glial endogenous cannabinoid system is upregulated in the brains of macaques with simian immunodeficiency virus-induced encephalitis. J. Neurosci..

[110] Xu J., Tang Y., Xie M., Bie B., Wu J., Yang H., Foss J.F., Yang B., Rosenquist R.W., Naguib M. (2016). Activation of cannabinoid receptor 2 attenuates mechanical allodynia and neuroinflammatory responses in a chronic post-ischemic pain model of complex regional pain syndrome type I in rats. Eur. J. Neurosci..

[111] Bie B., Wu J., Foss J.F., Naguib M. (2018). An overview of the cannabinoid type 2 receptor system and its therapeutic potential. Curr. Opin. Anaesthesiol..

[112] Benito C., Núñez E., Tolón R.M., Carrier E.J., Rábano A., Hillard C.J., Romero J. (2003). Cannabinoid CB2 Receptors and Fatty Acid Amide Hydrolase Are Selectively Overexpressed in Neuritic Plaque-Associated Glia in Alzheimer’s Disease Brains. J. Neurosci..

[113] Hossain M.Z., Unno S., Ando H., Masuda Y., Kitagawa J. (2017). Neuron-Glia crosstalk and neuropathic pain: Involvement in the modulation of motor activity in the Orofacial region. Int. J. Mol. Sci..

[114] Guo W., Wang H., Watanabe M., Shimizu K., Zou S., LaGraize S.C., Wei F., Dubner R., Ren K. (2007). Glial-cytokine-neuronal interactions underlying the mechanisms of persistent pain. J. Neurosci..

[115] Scholz J., Woolf C.J. (2007). The neuropathic pain triad: Neurons, immune cells and glia. Nat. Neurosci..

[116] Romero-Sandoval A., Nutile-Mcmenemy N., Deleo J.A. (2008). Spinal microglial and perivascular cell cannabinoid receptor type 2 activation reduces behavioral hypersensitivity without tolerance after peripheral nerve injury. Anesthesiology.

[117] Naguib M., Xu J.J., Diaz P., Brown D.L., Cogdell D., Bie B., Hu J., Craig S., Hittelman W.N. (2012). Prevention of paclitaxel-induced neuropathy through activation of the central cannabinoid type 2 receptor system. Anesth. Analg..

[118] Zhang J., Chen L., Su T., Cao F., Meng X., Pei L., Shi J., Pan H.L., Li M. (2010). Electroacupuncture increases CB2 receptor expression on keratinocytes and infiltrating inflammatory cells in inflamed skin tissues of rats. J. Pain.

[119] Bort A., Alvarado-Vazquez P.A., Moracho-Vilrriales C., Virga K.G., Gumina G., Romero-Sandoval A., Asbill S. (2017). Effects of JWH015 in cytokine secretion in primary human keratinocytes and fibroblasts and its suitability for topical/transdermal delivery. Mol. Pain.

[120] Turcotte C., Blanchet M.R., Laviolette M., Flamand N. (2016). The CB2 receptor and its role as a regulator of inflammation. Cell. Mol. Life Sci..

[121] Elmes S.J.R., Jhaveri M.D., Smart D., Kendall D.A., Chapman V. (2004). Cannabinoid CB2 receptor activation inhibits mechanically evoked responses of wide dynamic range dorsal horn neurons in naïve rats and in rat models of inflammatory and neuropathic pain. Eur. J. Neurosci..

[122] Clayton N., Marshall F.H., Bountra C., O’Shaughnessy C.T. (2002). CB1 and CB2 cannabinoid receptors are implicated in inflammatory pain. Pain.

[123] Hanuš L., Breuer A., Tchilibon S., Shiloah S., Goldenberg D., Horowitz M., Pertwee R.G., Ross R.A., Mechoulam R., Fride E. (1999). HU-308: A specific agonist for CB2, a peripheral cannabinoid receptor. Proc. Natl. Acad. Sci. USA.

[124] Malan T.P., Ibrahim M.M., Deng H., Makriyannis A., Vanderah T.W. (2001). Anti-inflammatory effects of the CB2 cannabinoid receptor-selective agonist AM1241. Anesthesiology.

[125] Quartilho A., Mata H.P., Ibrahim M.M., Vanderah T.W., Porreca F., Makriyannis A., Malan T.P. (2003). Inhibition of inflammatory hyperalgesia by activation of peripheral CB 2 cannabinoid receptors. Anesthesiology.

[126] Leichsenring A., Andriske M., Bäcker I., Stichel C.C., Lübbert H. (2009). Analgesic and antiinflammatory effects of cannabinoid receptor agonists in a rat model of neuropathic pain. Naunyn. Schmiedebergs. Arch. Pharmacol..

[127] Valenzano K.J., Tafesse L., Lee G., Harrison J.E., Boulet J.M., Gottshall S.L., Mark L., Pearson M.S., Miller W., Shan S. (2005). Pharmacological and pharmacokinetic characterization of the cannabinoid receptor 2 agonist, GW405833, utilizing rodent models of acute and chronic pain, anxiety, ataxia and catalepsy. Neuropharmacology.

[128] LaBuda C.J., Koblish M., Little P.J. (2005). Cannabinoid CB2 receptor agonist activity in the hindpaw incision: Model of postoperative pain. Eur. J. Pharmacol..

[129] Whiteside G.T., Gottshall S.L., Boulet J.M., Chaffer S.M., Harrison J.E., Pearson M.S., Turchin P.I., Mark L., Garrison A.E., Valenzano K.J. (2005). A role for cannabinoid receptors, but not endogenous opioids, in the antinociceptive activity of the CB2-selective agonist, GW405833. Eur. J. Pharmacol..

[130] Pasquini S., Mugnaini C., Ligresti A., Tafi A., Brogi S., Falciani C., Pedani V., Pesco N., Guida F., Luongo L. (2012). Design, synthesis, and pharmacological characterization of indol-3-ylacetamides, indol-3-yloxoacetamides, and indol-3-ylcarboxamides: Potent and selective CB2 cannabinoid receptor inverse agonists. J. Med. Chem..

[131] Hervera A., Negrete R., Leánez S., Martín-Campos J., Pol O. (2010). The role of nitric oxide in the local antiallodynic and antihyperalgesic effects and expression of δ-opioid and cannabinoid-2 receptors during neuropathic pain in mice. J. Pharmacol. Exp. Ther..

[132] Castany S., Carcolé M., Leánez S., Pol O. (2016). The role of carbon monoxide on the anti-nociceptive effects and expression of cannabinoid 2 receptors during painful diabetic neuropathy in mice. Psychopharmacology.

[133] Rahn E.J., Makriyannis A., Hohmann A.G. (2007). Activation of cannabinoid CB 1 and CB 2 receptors suppresses neuropathic nociception evoked by the chemotherapeutic agent vincristine in rats. Br. J. Pharmacol..

[134] Rahn E.J., Zvonok A.M., Thakur G.A., Khanolkar A.D., Makriyannis A., Hohmann A.G. (2008). Selective activation of cannabinoid CB2 receptors suppresses neuropathic nociception induced by treatment with the chemotherapeutic agent paclitaxel in rats. J. Pharmacol. Exp. Ther..

[135] Bujalska M. (2008). Effect of cannabinoid receptor agonists on streptozotocin-induced hyperalgesia in diabetic neuropathy. Pharmacology.

[136] Bujalska-Zadrozny M., De Cordé A., Pawlik K. (2015). Influence of nitric oxide synthase or cyclooxygenase inhibitors on cannabinoids activity in streptozotocin-induced neuropathy. Pharmacol. Reports.

[137] Gutierrez T., Crystal J.D., Zvonok A.M., Makriyannis A., Hohmann A.G. (2011). Self-medication of a cannabinoid CB 2 agonist in an animal model of neuropathic pain. Pain.

[138] Wilkerson J.L., Gentry K.R., Dengler E.C., Wallace J.A., Kerwin A.A., Kuhn M.N., Zvonok A.M., Thakur G.A., Makriyannis A., Milligan E.D. (2012). Immunofluorescent spectral analysis reveals the intrathecal cannabinoid agonist, AM1241, produces spinal anti-inflammatory cytokine responses in neuropathic rats exhibiting relief from allodynia. Brain Behav..

[139] Wilkerson J.L., Gentry K.R., Dengler E.C., Wallace J.A., Kerwin A.A., Armijo L.M., Kuhn M.N., Thakur G.A., Makriyannis A., Milligan E.D. (2012). Intrathecal cannabilactone CB 2R agonist, AM1710, controls pathological pain and restores basal cytokine levels. Pain.

[140] Hu B., Doods H., Treede R.D., Ceci A. (2009). Depression-like behaviour in rats with mononeuropathy is reduced by the CB2-selective agonist GW405833. Pain.

[141] Naguib M., Diaz P., Xu J.J., Astruc-Diaz F., Craig S., Vivas-Mejia P., Brown D.L. (2008). MDA7: A novel selective agonist for CB 2 receptors that prevents allodynia in rat neuropathic pain models. Br. J. Pharmacol..

[142] Xu J.J., Diaz P., Astruc-Diaz F., Craig S., Munoz E., Naguib M. (2010). Pharmacological characterization of a novel cannabinoid ligand, MDA19, for treatment of neuropathic pain. Anesth. Analg..

[143] Luongo L., Palazzo E., Tambaro S., Giordano C., Gatta L., Scafuro M.A., sca Rossi F., Lazzari P., Pani L., de Novellis V. (2010). 1-(2′,4′-dichlorophenyl)-6-methyl-N-cyclohexylamine-1,4-dihy droindeno[1,2-c]pyrazole-3-carboxamide, a novel CB2 agonist, alleviates neuropathic pain through functional microglial changes in mice. Neurobiol. Dis..

[144] Yamamoto W., Mikami T., Iwamura H. (2008). Involvement of central cannabinoid CB2 receptor in reducing mechanical allodynia in a mouse model of neuropathic pain. Eur. J. Pharmacol..

[145] Deng L., Guindon J., Vemuri V.K., Thakur G.A., White F.A., Makriyannis A., Hohmann A.G. (2012). The maintenance of cisplatin- and paclitaxel-induced mechanical and cold allodynia is suppressed by cannabinoid CB2 receptor activation and independent of CXCR4 signaling in models of chemotherapy-induced peripheral neuropathy. Mol. Pain.

[146] Rahn E.J., Deng L., Thakur G.A., Vemuri K., Zvonok A.M., Lai Y.Y., Makriyannis A., Hohmann A.G. (2014). Prophylactic cannabinoid administration blocks the development of paclitaxel-induced neuropathic nociception during analgesic treatment and following cessation of drug delivery. Mol. Pain.

[147] Deng L., Guindon J., Cornett B.L., Makriyannis A., Mackie K., Hohmann A.G. (2015). Chronic cannabinoid receptor 2 activation reverses paclitaxel neuropathy without tolerance or cannabinoid receptor 1-dependent withdrawal. Biol. Psychiatry.

[148] Ikeda H., Ikegami M., Kai M., Ohsawa M., Kamei J. (2013). Activation of spinal cannabinoid CB2 receptors inhibits neuropathic pain in streptozotocin-induced diabetic mice. Neuroscience.

[149] Lin X., Dhopeshwarkar A.S., Huibregtse M., MacKie K., Hohmann A.G. (2018). Slowly signaling G protein-biased CB2 cannabinoid receptor agonist LY2828360 suppresses neuropathic pain with sustained efficacy and attenuates morphine tolerance and dependence. Mol. Pharmacol..

[150] Malan T.P., Ibrahim M.M., Deng H., Liu Q., Mata H.P., Vanderah T., Porreca F., Makriyannis A. (2001). CB2 cannabinoid receptor-mediated peripheral antinociception. Pain.

[151] Hohmann A.G., Farthing J.N., Zvonok A.M., Makriyannis A. (2004). Selective Activation of Cannabinoid CB2 Receptors Suppresses Hyperalgesia Evoked by Intradermal Capsaicin. J. Pharmacol. Exp. Ther..

[152] Rahn E.J., Thakur G.A., Wood J.A.T., Zvonok A.M., Makriyannis A., Hohmann A.G. (2011). Pharmacological characterization of AM1710, a putative cannabinoid CB 2 agonist from the cannabilactone class: Antinociception without central nervous system side-effects. Pharmacol. Biochem. Behav..

[153] Xu J.J., Diaz P., Bie B., Astruc-Diaz F., Wu J., Yang H., Brown D.L., Naguib M. (2014). Spinal gene expression profiling and pathways analysis of a CB2 agonist (MDA7)-targeted prevention of paclitaxel-induced neuropathy. Neuroscience.

[154] Patel H.J., Birrell M.A., Crispino N., Hele D.J., Venkatesan P., Barnes P.J., Yacoub M.H., Belvisi M.G. (2003). Inhibition of guinea-pig and human sensory nerve activity and the cough reflex in guinea-pigs by cannabinoid (CB 2) receptor activation. Br. J. Pharmacol..

[155] Ross R.A., Coutts A.A., McFarlane S.M., Anavi-Goffer S., Irving A.J., Pertwee R.G., MacEwan D.J., Scott R.H. (2001). Actions of cannabinoid receptor ligands on rat cultured sensory neurones: Implications for antinociception. Neuropharmacology.

[156] Schmid H.H.O. (2000). Pathways and mechanisms of N-acylethanolamine biosynthesis: Can anandamide be generated selectively?. Chem. Phys. Lipids.

[157] Pertwee R.G. (2015). Endocannabinoids and their pharmacological actions. Handbook of Experimental Pharmacology.

[158] Di Marzo V. (2018). New approaches and challenges to targeting the endocannabinoid system. Nat. Rev. Drug Discov..

[159] Stella N., Schweitzer P., Plomelli D. (1997). A second endogenous’ cannabinoid that modulates long-term potentiation. Nature.

[160] Pertwee R. (2010). Receptors and Channels Targeted by Synthetic Cannabinoid Receptor Agonists and Antagonists. Curr. Med. Chem..

[161] Wilson R.I., Nicoll R.A. (2001). Endogenous cannabinoids mediate retrograde signalling at hippocampal synapses. Nature.

[162] Ohno-Shosaku T., Maejima T., Kano M. (2001). Endogenous cannabinoids mediate retrograde signals from depolarized postsynaptic neurons to presynaptic terminals. Neuron.

[163] Kreitzer A.C., Regehr W.G. (2001). Retrograde inhibition of presynaptic calcium influx by endogenous cannabinoids at excitatory synapses onto Purkinje cells. Neuron.

[164] Cravatt B.F., Giang D.K., Mayfield S.P., Boger D.L., Lerner R.A., Gilula N.B. (1996). Molecular characterization of an enzyme that degrades neuromodulatory fatty-acid amides. Nature.

[165] Blankman J.L., Simon G.M., Cravatt B.F. (2007). A Comprehensive Profile of Brain Enzymes that Hydrolyze the Endocannabinoid 2-Arachidonoylglycerol. Chem. Biol..

[166] Fowler C.J. (2007). The contribution of cyclooxygenase-2 to endocannabinoid metabolism and action. Br. J. Pharmacol..

[167] Calignano A., La Rana G., Giuffrida A., Piomelli D. (1998). Control of pain initiation by endogenous cannabinoids. Nature.

[168] Strangman N.M., Patrick S.L., Hohmann A.G., Tsou K., Walker J.M. (1998). Evidence for a role of endogenous cannabinoids in the modulation of acute and tonic pain sensitivity. Brain Res..

[169] Smith P.B., Compton D.R., Welch S.P., Razdan R.K., Mechoulam R., Martin B.R. (1994). The pharmacological activity of anandamide, a putative endogenous cannabinoid, in mice. J. Pharmacol. Exp. Ther..

[170] Smith S.C., Wagner M.S. (2014). Clinical endocannabinoid deficiency (CECD) revisited: Can this concept explain the therapeutic benefits of cannabis in migraine, fibromyalgia, irritable bowel syndrome and other treatment-resistant conditions?. Neuroendocrinol. Lett..

[171] Russo E.B. (2016). Clinical Endocannabinoid Deficiency Reconsidered: Current Research Supports the Theory in Migraine, Fibromyalgia, Irritable Bowel, and Other Treatment-Resistant Syndromes. Cannabis Cannabinoid Res..

[172] Masocha W. (2018). Targeting the Endocannabinoid System for Prevention or Treatment of Chemotherapy-Induced Neuropathic Pain: Studies in Animal Models. Pain Res. Manag..

[173] Pandey R., Mousawy K., Nagarkatti M., Nagarkatti P. (2009). Endocannabinoids and immune regulation. Pharmacol. Res..

[174] Pestonjamasp V.K., Burstein S.H. (1998). Anandamide synthesis is induced by arachidonate mobilizing agonists in cells of the immune system. Biochim. Biophys. Acta Lipids Lipid Metab..

[175] Bisogno T., Maurelli S., Melck D., De Petrocellis L., Di Marzo V. (1997). Biosynthesis, uptake, and degradation of anandamide and palmitoylethanolamide in leukocytes. J. Biol. Chem..

[176] Stella N. (2009). Endocannabinoid signaling in microglial cells. Neuropharmacology.

[177] Mecha M., Feliú A., Carrillo-Salinas F.J., Rueda-Zubiaurre A., Ortega-Gutiérrez S., de Sola R.G., Guaza C. (2015). Endocannabinoids drive the acquisition of an alternative phenotype in microglia. Brain. Behav. Immun..

[178] Mecha M., Carrillo-Salinas F.J., Feliú A., Mestre L., Guaza C. (2016). Microglia activation states and cannabinoid system: Therapeutic implications. Pharmacol. Ther..

[179] Shinoda M., Kubo A., Hayashi Y., Iwata K. (2019). Peripheral and Central Mechanisms of Persistent Orofacial Pain. Front. Neurosci..

[180] Petrosino S., Palazzo E., de Novellis V., Bisogno T., Rossi F., Maione S., Di Marzo V. (2007). Changes in spinal and supraspinal endocannabinoid levels in neuropathic rats. Neuropharmacology.

[181] Guasti L., Richardson D., Jhaveri M., Eldeeb K., Barrett D., Elphick M.R., Alexander S.P.H., Kendall D., Michael G.J., Chapman V. (2009). Minocycline Treatment Inhibits Microglial Activation and Alters Spinal Levels of Endocannabinoids in a Rat Model of Neuropathic Pain. Mol. Pain.

[182] Giordano C., Cristino L., Luongo L., Siniscalco D., Petrosino S., Piscitelli F., Marabese I., Gatta L., Rossi F., Imperatore R. (2012). TRPV1-dependent and-independent alterations in the limbic cortex of neuropathic mice: Impact on glial caspases and pain perception. Cereb. Cortex.

[183] Guindon J., Lai Y., Takacs S.M., Bradshaw H.B., Hohmann A.G. (2013). Alterations in endocannabinoid tone following chemotherapy-induced peripheral neuropathy: Effects of endocannabinoid deactivation inhibitors targeting fatty-acid amide hydrolase and monoacylglycerol lipase in comparison to reference analgesics following cisplatin treatment. Pharmacol. Res..

[184] Palazzo E., De Novellis V., Petrosino S., Marabese I., Vita D., Giordano C., Di Marzo V., Mangoni G.S., Rossi F., Maione S. (2006). Neuropathic pain and the endocannabinoid system in the dorsal raphe: Pharmacological treatment and interactions with the serotonergic system. Eur. J. Neurosci..

[185] Walker J.M., Huang S.M., Strangman N.M., Tsou K., Sañudo-Peña M.C. (1999). Pain modulation by release of the endogenous cannabinoid anandamide. Proc. Natl. Acad. Sci. USA.

[186] Guindon J., Desroches J., Beaulieu P. (2007). The antinociceptive effects of intraplantar injections of 2-arachidonoyl glycerol are mediated by cannabinoid CB 2 receptors. Br. J. Pharmacol..

[187] Jhaveri M.D., Richardson D., Kendall D.A., Barrett D.A., Chapman V. (2006). Analgesic effects of fatty acid amide hydrolase inhibition in a rat model of neuropathic pain. J. Neurosci..

[188] D’Argenio G., Valenti M., Scaglione G., Cosenza V., Sorrentini I., Di Marzo V. (2006). Di Up-regulation of anandamide levels as an endogenous mechanism and a pharmacological strategy to limit colon inflammation. FASEB J..

[189] Sokal D.M., Elmes S.J.R., Kendall D.A., Chapman V. (2003). Intraplantar injection of anandamide inhibits mechanically-evoked responses of spinal neurones via activation of CB2 receptors in anaesthetised rats. Neuropharmacology.

[190] Jardín I., López J.J., Diez R., Sánchez-Collado J., Cantonero C., Albarrán L., Woodard G.E., Redondo P.C., Salido G.M., Smani T. (2017). TRPs in pain sensation. Front. Physiol..

[191] Hossain M.Z., Bakri M.M., Yahya F., Ando H., Unno S., Kitagawa J. (2019). The role of transient receptor potential (TRP) channels in the transduction of dental pain. Int. J. Mol. Sci..

[192] Marrone M.C., Morabito A., Giustizieri M., Chiurchiù V., Leuti A., Mattioli M., Marinelli S., Riganti L., Lombardi M., Murana E. (2017). TRPV1 channels are critical brain inflammation detectors and neuropathic pain biomarkers in mice. Nat. Commun..

[193] Vyklický L., Nováková-Toušová K., Benedikt J., Samad A., Touška F., Vlachova V. (2008). Calcium-dependent desensitization of vanilloid receptor TRPV1: A mechanism possibly involved in analgesia induced by topical application of capsaicin. Physiol. Res..

[194] Fride E., Mechoulam R. (1993). Pharmacological activity of the cannabinoid receptor agonist, anandamide, a brain constituent. Eur. J. Pharmacol..

[195] Jaggar S.I., Hasnie F.S., Sellaturay S., Rice A.S.C. (1998). The anti-hyperalgesic actions of the cannabinoid anandamide and the putative CB2 receptor agonist palmitoylethanolamide in visceral and somatic inflammatory pain. Pain.

[196] Kathuria S., Gaetani S., Fegley D., Valiño F., Duranti A., Tontini A., Mor M., Tarzia G., La Rana G., Calignano A. (2003). Modulation of anxiety through blockade of anandamide hydrolysis. Nat. Med..

[197] Long J.Z., Li W., Booker L., Burston J.J., Kinsey S.G., Schlosburg J.E., Pavón F.J., Serrano A.M., Selley D.E., Parsons L.H. (2009). Selective blockade of 2-arachidonoylglycerol hydrolysis produces cannabinoid behavioral effects. Nat. Chem. Biol..

[198] Ahn K., Smith S.E., Liimatta M.B., Beidler D., Sadagopan N., Dudley D.T., Young T., Wren P., Zhang Y., Swaney S. (2011). Mechanistic and pharmacological characterization of PF-04457845: A highly potent and selective fatty acid amide hydrolase inhibitor that reduces inflammatory and noninflammatory pain. J. Pharmacol. Exp. Ther..

[199] Kinsey S.G., Long J.Z., O’Neal S.T., Abdullah R.A., Poklis J.L., Boger D.L., Cravatt B.F., Lichtman A.H. (2009). Blockade of endocannabinoid-degrading enzymes attenuates neuropathic pain. J. Pharmacol. Exp. Ther..

[200] Caprioli A., Coccurello R., Rapino C., Di Serio S., Di Tommaso M., Vertechy M., Vacca V., Battista N., Pavone F., Maccarrone M. (2012). The novel reversible fatty acid amide hydrolase inhibitor ST4070 increases endocannabinoid brain levels and counteracts neuropathic pain in different animal models. J. Pharmacol. Exp. Ther..

[201] Adamson Barnes N.S., Mitchell V.A., Kazantzis N.P., Vaughan C.W. (2016). Actions of the dual FAAH/MAGL inhibitor JZL195 in a murine neuropathic pain model. Br. J. Pharmacol..

[202] Russo R., LoVerme J., La Rana G., Compton T.R., Parrott J., Duranti A., Tontini A., Mor M., Tarzia G., Calignano A. (2007). The fatty acid amide hydrolase inhibitor URB597 (cyclohexylcarbamic acid 3′-carbamoylbiphenyl-3-yl ester) reduces neuropathic pain after oral administration in mice. J. Pharmacol. Exp. Ther..

[203] Starowicz K., Makuch W., Osikowicz M., Piscitelli F., Petrosino S., Di Marzo V., Przewlocka B. (2012). Spinal anandamide produces analgesia in neuropathic rats: Possible CB 1- and TRPV1-mediated mechanisms. Neuropharmacology.

[204] Starowicz K., Makuch W., Korostynski M., Malek N., Slezak M., Zychowska M., Petrosino S., De Petrocellis L., Cristino L., Przewlocka B. (2013). Full Inhibition of Spinal FAAH Leads to TRPV1-Mediated Analgesic Effects in Neuropathic Rats and Possible Lipoxygenase-Mediated Remodeling of Anandamide Metabolism. PLoS ONE.

[205] Desroches J., Charron S., Bouchard J.F., Beaulieu P. (2014). Endocannabinoids decrease neuropathic pain-Related behavior in mice through the activation of one or both peripheral CB1 and CB2 receptors. Neuropharmacology.

[206] Desroches J., Guindon J., Lambert C., Beaulieu P. (2008). Modulation of the anti-nociceptive effects of 2-arachidonoyl glycerol by peripherally administered FAAH and MGL inhibitors in a neuropathic pain model. Br. J. Pharmacol..

[207] Malek N., Kostrzewa M., Makuch W., Pajak A., Kucharczyk M., Piscitelli F., Przewlocka B., Di Marzo V., Starowicz K. (2016). The multiplicity of spinal AA-5-HT anti-nociceptive action in a rat model of neuropathic pain. Pharmacol. Res..

[208] de Novellis V., Vita D., Gatta L., Luongo L., Bellini G., De Chiaro M., Marabese I., Siniscalco D., Boccella S., Piscitelli F. (2011). The blockade of the transient receptor potential vanilloid type 1 and fatty acid amide hydrolase decreases symptoms and central sequelae in the medial prefrontal cortex of neuropathic rats. Mol. Pain.

[209] Slivicki R.A., Xu Z., Kulkarni P.M., Pertwee R.G., Mackie K., Thakur G.A., Hohmann A.G. (2018). Positive Allosteric Modulation of Cannabinoid Receptor Type 1 Suppresses Pathological Pain Without Producing Tolerance or Dependence. Biol. Psychiatry.

[210] Slivicki R.A., Saberi S.A., Iyer V., Vemuri V.K., Makriyannis A., Hohmann A.G. (2018). Brain-permeant and -impermeant inhibitors of fatty acid amide hydrolase synergize with the opioid analgesic morphine to suppress chemotherapy-induced neuropathic nociception without enhancing effects of morphine on gastrointestinal transit. J. Pharmacol. Exp. Ther..

[211] Niphakis M.J., Cognetta A.B., Chang J.W., Buczynski M.W., Parsons L.H., Byrne F., Burston J.J., Chapman V., Cravatt B.F. (2013). Evaluation of NHS carbamates as a potent and selective class of endocannabinoid hydrolase inhibitors. ACS Chem. Neurosci..

[212] Ghosh S., Kinsey S.G., Liu Q.S., Hruba L., McMahon L.R., Grim T.W., Merritt C.R., Wise L.E., Abdullah R.A., Selley D.E. (2015). Full fatty acid amide hydrolase inhibition combined with partial monoacylglycerol lipase inhibition: Augmented and sustained antinociceptive effects with reduced cannabimimetic side effects in mice. J. Pharmacol. Exp. Ther..

[213] Grim T.W., Ghosh S., Hsu K.L., Cravatt B.F., Kinsey S.G., Lichtman A.H. (2014). Combined inhibition of FAAH and COX produces enhanced anti-allodynic effects in mouse neuropathic and inflammatory pain models. Pharmacol. Biochem. Behav..

[214] Schlosburg J.E., Blankman J.L., Long J.Z., Nomura D.K., Pan B., Kinsey S.G., Nguyen P.T., Ramesh D., Booker L., Burston J.J. (2010). Chronic monoacylglycerol lipase blockade causes functional antagonism of the endocannabinoid system. Nat. Neurosci..

[215] Kinsey S.G., Long J.Z., Cravatt B.F., Lichtman A.H. (2010). Fatty acid amide hydrolase and monoacylglycerol lipase inhibitors produce anti-allodynic effects in mice through distinct cannabinoid receptor mechanisms. J. Pain.

[216] Clapper J.R., Moreno-Sanz G., Russo R., Guijarro A., Vacondio F., Duranti A., Tontini A., Sanchini S., Sciolino N.R., Spradley J.M. (2010). Anandamide suppresses pain initiation through a peripheral endocannabinoid mechanism. Nat. Neurosci..

[217] Sasso O., Bertorelli R., Bandiera T., Scarpelli R., Colombano G., Armirotti A., Moreno-Sanz G., Reggiani A., Piomelli D. (2012). Peripheral FAAH inhibition causes profound antinociception and protects against indomethacin-induced gastric lesions. Pharmacol. Res..

[218] Jayamanne A., Greenwood R., Mitchell V.A., Aslan S., Piomelli D., Vaughan C.W. (2006). Actions of the FAAH inhibitor URB597 in neuropathic and inflammatory chronic pain models. Br. J. Pharmacol..

[219] Ahn K., Johnson D.S., Mileni M., Beidler D., Long J.Z., McKinney M.K., Weerapana E., Sadagopan N., Liimatta M., Smith S.E. (2009). Discovery and Characterization of a Highly Selective FAAH Inhibitor that Reduces Inflammatory Pain. Chem. Biol..

[220] Ghosh S., Wise L.E., Chen Y., Gujjar R., Mahadevan A., Cravatt B.F., Lichtman A.H. (2013). The monoacylglycerol lipase inhibitor JZL184 suppresses inflammatory pain in the mouse carrageenan model. Life Sci..

[221] Maione S., Morera E., Marabese I., Ligresti A., Luongo L., Ortar G., Di Marzo V. (2008). Antinociceptive effects of tetrazole inhibitors of endocannabinoid inactivation: Cannabinoid and non-cannabinoid receptor-mediated mechanisms. Br. J. Pharmacol..

[222] Naidu P.S., Booker L., Cravatt B.F., Lichtman A.H. (2009). Synergy between enzyme inhibitors of fatty acid amide hydrolase and cyclooxygenase in visceral nociception. J. Pharmacol. Exp. Ther..

[223] Kinsey S.G., Wise L.E., Ramesh D., Abdullah R., Selley D.E., Cravatt B.F., Lichtman A.H. (2013). Repeated low-dose administration of the monoacylglycerol lipase inhibitor JZL184 retains cannabinoid receptor type 1-mediated antinociceptive and gastroprotective effects. J. Pharmacol. Exp. Ther..

[224] Ignatowska-Jankowska B.M., Ghosh S., Crowe M.S., Kinsey S.G., Niphakis M.J., Abdullah R.A., Tao Q., O’Neal S.T., Walentiny D.M., Wiley J.L. (2014). In vivo characterization of the highly selective monoacylglycerol lipase inhibitor KML29: Antinociceptive activity without cannabimimetic side effects. Br. J. Pharmacol..

[225] Crowe M.S., Leishman E., Banks M.L., Gujjar R., Mahadevan A., Bradshaw H.B., Kinsey S.G. (2015). Combined inhibition of monoacylglycerol lipase and cyclooxygenases synergistically reduces neuropathic pain in mice. Br. J. Pharmacol..

[226] Ignatowska-Jankowska B., Wilkerson J.L., Mustafa M., Abdullah R., Niphakis M., Wiley J.L., Cravatt B.F., Lichtman A.H. (2015). Selective monoacylglycerol lipase inhibitors: Antinociceptive versus cannabimimetic effects in Mices. J. Pharmacol. Exp. Ther..

[227] Curry Z.A., Wilkerson J.L., Bagdas D., Kyte S.L., Patel N., Donvito G., Mustafa M.A., Poklis J.L., Niphakis M.J., Hsu K.L. (2018). Monoacylglycerol lipase inhibitors reverse paclitaxel-induced nociceptive behavior and proinflammatory markers in a mouse model of chemotherapy-induced neuropathy. J. Pharmacol. Exp. Ther..

[228] Khasabova I.A., Yao X., Paz J., Lewandowski C.T., Lindberg A.E., Coicou L., Burlakova N., Simone D.A., Seybold V.S. (2014). JZL184 is anti-hyperalgesic in a murine model of cisplatin-induced peripheral neuropathy. Pharmacol. Res..

[229] Makara J.K., Mor M., Fegley D., Szabó S.I., Kathuria S., Astarita G., Duranti A., Tontini A., Tarzia G., Rivara S. (2005). Selective inhibition of 2-AG hydrolysis enhances endocannabinoid signaling in hippocampus. Nat. Neurosci..

[230] Saario S.M., Salo O.M.H., Nevalainen T., Poso A., Laitinen J.T., Järvinen T., Niemi R. (2005). Characterization of the sulfhydryl-sensitive site in the enzyme responsible for hydrolysis of 2-arachidonoyl-glycerol in rat cerebellar membranes. Chem. Biol..

[231] Burston J.J., Sim-Selley L.J., Harloe J.P., Mahadevan A., Razdan R.K., Selley D.E., Wiley J.L. (2008). N-arachidonyl maleimide potentiates the pharmacological and biochemical effects of the endocannabinoid 2-arachidonylglycerol through inhibition of monoacylglycerol lipase. J. Pharmacol. Exp. Ther..

[232] Vandevoorde S., Jonsson K.O., Labar G., Persson E., Lambert D.M., Fowler C.J. (2007). Lack of selectivity of URB602 for 2-oleoylglycerol compared to anandamide hydrolysis in vitro. Br. J. Pharmacol..

[233] Wiskerke J., Irimia C., Cravatt B.F., De Vries T.J., Schoffelmeer A.N.M., Pattij T., Parsons L.H. (2012). Characterization of the effects of reuptake and hydrolysis inhibition on interstitial endocannabinoid levels in the brain: An in vivo microdialysis study. ACS Chem. Neurosci..

[234] Hohmann A.G., Suplita R.L., Bolton N.M., Neely M.H., Fegley D., Mangieri R., Krey J.F., Walker J.M., Holmes P.V., Crystal J.D. (2005). An endocannabinoid mechanism for stress-induced analgesia. Nature.

[235] Guindon J., Guijarro A., Piomelli D., Hohmann A.G. (2011). Peripheral antinociceptive effects of inhibitors of monoacylglycerol lipase in a rat model of inflammatory pain. Br. J. Pharmacol..

[236] Woodhams S.G., Wong A., Barrett D.A., Bennett A.J., Chapman V., Alexander S.P.H. (2012). Spinal administration of the monoacylglycerol lipase inhibitor JZL184 produces robust inhibitory effects on nociceptive processing and the development of central sensitization in the rat. Br. J. Pharmacol..

[237] Spradley J.M., Guindon J., Hohmann A.G. (2010). Inhibitors of monoacylglycerol lipase, fatty-acid amide hydrolase and endocannabinoid transport differentially suppress capsaicin-induced behavioral sensitization through peripheral endocannabinoid mechanisms. Pharmacol. Res..

[238] Wilkerson J.L., Ghosh S., Mustafa M., Abdullah R.A., Niphakis M.J., Cabrera R., Maldonado R., Cravatt B.F., Lichtman A.H. (2017). The endocannabinoid hydrolysis inhibitor SA-57: Intrinsic antinociceptive effects, augmented morphine-induced antinociception, and attenuated heroin seeking behavior in mice. Neuropharmacology.

[239] Anderson W.B., Gould M.J., Torres R.D., Mitchell V.A., Vaughan C.W. (2014). Actions of the dual FAAH/MAGL inhibitor JZL195 in a murine inflammatory pain model. Neuropharmacology.

[240] Sakin Y.S., Dogrul A., Ilkaya F., Seyrek M., Ulas U.H., Gulsen M., Bagci S. (2015). The effect of FAAH, MAGL, and Dual FAAH/MAGL inhibition on inflammatory and colorectal distension-induced visceral pain models in Rodents. Neurogastroenterol. Motil..

[241] Long J.Z., Nomura D.K., Vann R.E., Walentiny D.M., Booker L., Jin X., Burston J.J., Sim-Selley L.J., Lichtman A.H., Wiley J.L. (2009). Dual blockade of FAAH and MAGL identifies behavioral processes regulated by endocannabinoid crosstalk in vivo. Proc. Natl. Acad. Sci. USA.

[242] Wilkerson J.L., Niphakis M.J., Grim T.W., Mustafa M.A., Abdullah R.A., Poklis J.L., Dewey W.L., Akbarali H., Banks M.L., Wise L.E. (2016). The selective monoacylglycerol lipase inhibitor MJN110 produces opioid-sparing effects in a mouse neuropathic pain model. J. Pharmacol. Exp. Ther..

[243] Sessle B.J. (2015). Editorial: Are Cannabinoids Effective for Orofacial Pain States?. J. Oral Facial Pain Headache.

[244] McDonough P., McKenna J.P., McCreary C., Downer E.J. (2014). Neuropathic orofacial pain: Cannabinoids as a therapeutic avenue. Int. J. Biochem. Cell Biol..

[245] Nakajima Y., Furuichi Y., Biswas K.K., Hashiguchi T., Kawahara K.I., Yamaji K., Uchimura T., Izumi Y., Maruyama I. (2006). Endocannabinoid, anandamide in gingival tissue regulates the periodontal inflammation through NF-κB pathway inhibition. FEBS Lett..

[246] Borsani E., Majorana A., Cocchi M.A., Conti G., Bonadeo S., Padovani A., Lauria G., Bardellini E., Rezzani R., Rodella L.F. (2014). Epithelial expression of vanilloid and cannabinoid receptors: A potential role in burning mouth syndrome pathogenesis. Histol. Histopathol..

[247] Cupini L.M., Costa C., Sarchielli P., Bari M., Battista N., Eusebi P., Calabresi P., Maccarrone M. (2008). Degradation of endocannabinoids in chronic migraine and medication overuse headache. Neurobiol. Dis..

[248] Nozaki C., Markert A., Zimmer A. (2015). Inhibition of FAAH reduces nitroglycerin-induced migraine-like pain and trigeminal neuronal hyperactivity in mice. Eur. Neuropsychopharmacol..

[249] Greco R., Gasperi V., Sandrini G., Bagetta G., Nappi G., MacCarrone M., Tassorelli C. (2010). Alterations of the endocannabinoid system in an animal model of migraine: Evaluation in cerebral areas of rat. Cephalalgia.

[250] Liang Y.C., Huang C.C., Hsu K.S., Takahashi T. (2004). Cannabinoid-induced presynaptic inhibition at the primary afferent trigeminal synapse of juvenile rat brainstem slices. J. Physiol..

[251] Papanastassiou A.M., Fields H.L., Meng I.D. (2004). Local application of the cannabinoid receptor agonist, WIN 55,212-2, to spinal trigeminal nucleus caudalis differentially affects nociceptive and non-nociceptive neurons. Pain.

[252] Li Z.W., Zhang J., Ouyang C.H., Li C.Y., Zhao F.B., Liu Y.W., Ai Y.X., Hu W.P. (2009). Potentiation by WIN 55,212-2 of GABA-activated currents in rat trigeminal ganglion neurones. Br. J. Pharmacol..

[253] Shi B., Yang R., Wang X., Liu H., Zou L., Hu X., Wu J., Zou A., Liu L. (2012). Inhibition of 5-HT3 receptors-activated currents by cannabinoids in rat trigeminal ganglion neurons. J. Huazhong Univ. Sci. Technol. Med. Sci..

[254] Wang W., Cao X., Liu C., Liu L. (2012). Cannabinoid WIN 55,212-2 inhibits TRPV1 in trigeminal ganglion neurons via PKA and PKC pathways. Neurol. Sci..

[255] Price T.J., Patwardhan A., Akopian A.N., Hargreaves K.M., Flores C.M. (2004). Modulation of trigeminal sensory neuron activity by the dual cannabinoid-vanilloid agonists anandamide, N-arachidonoyl-dopamine and arachidonyl-2-chloroethylamide. Br. J. Pharmacol..

[256] Greco R., Mangione A.S., Sandrini G., Nappi G., Tassorelli C. (2014). Activation of CB2 receptors as a potential therapeutic target for migraine: Evaluation in an animal model. J. Headache Pain.

[257] Greco R., Bandiera T., Mangione A.S., Demartini C., Siani F., Nappi G., Sandrini G., Guijarro A., Armirotti A., Piomelli D. (2015). Effects of peripheral FAAH blockade on NTG-induced hyperalgesia—Evaluation of URB937 in an animal model of migraine. Cephalalgia.

[258] Kamimura R., Hossain M.Z., Unno S., Ando H., Masuda Y., Takahashi K., Otake M., Saito I., Kitagawa J. (2018). Inhibition of 2-arachydonoylgycerol degradation attenuates orofacial neuropathic pain in trigeminal nerve-injured mice. J. Oral Sci..

[259] Leimuranta P., Khiroug L., Giniatullin R. (2018). Emerging role of (endo)cannabinoids in migraine. Front. Pharmacol..

[260] Akerman S., Holland P.R., Goadsby P.J. (2007). Cannabinoid (CB1) receptor activation inhibits trigeminovascular neurons. J. Pharmacol. Exp. Ther..

[261] Akerman S., Holland P.R., Lasalandra M.P., Goadsby P.J. (2013). Endocannabinoids in the brainstem modulate dural trigeminovascular nociceptive traffic via CB1 and “Triptan” receptors: Implications in migraine. J. Neurosci..

[262] Burgos E., Pascual D., Isabel Martín M., Goicoechea C. (2010). Antinociceptive effect of the cannabinoid agonist, WIN 55,212-2, in the orofacial and temporomandibular formalin tests. Eur. J. Pain.

[263] Mostafeezur R.M., Shinoda M., Unno S., Zakir H.M., Takatsuji H., Takahashi K., Yamada Y., Yamamura K., Iwata K., Kitagawa J. (2014). Involvement of astroglial glutamate-glutamine shuttle in modulation of the jaw-opening reflex following infraorbital nerve injury. Eur. J. Neurosci..

[264] Mostafeezur R.M., Zakir H.M., Yamada Y., Yamamura K., Iwata K., Sessle B.J., Kitagawa J. (2012). The effect of minocycline on the masticatory movements following the inferior alveolar nerve transection in freely moving rats. Mol. Pain.

[265] Zakir H.M., Mostafeezur R.M., Suzuki A., Hitomi S., Suzuki I., Maeda T., Seo K., Yamada Y., Yamamura K., Lev S. (2012). Expression of TRPV1 Channels after Nerve Injury Provides an Essential Delivery Tool for Neuropathic Pain Attenuation. PLoS ONE.

[266] Mátyás F., Urbán G.M., Watanabe M., Mackie K., Zimmer A., Freund T.F., Katona I. (2008). Identification of the sites of 2-arachidonoylglycerol synthesis and action imply retrograde endocannabinoid signaling at both GABAergic and glutamatergic synapses in the ventral tegmental area. Neuropharmacology.

[267] Katona I., Freund T.F. (2008). Endocannabinoid signaling as a synaptic circuit breaker in neurological disease. Nat. Med..

[268] Navarro G., Borroto-Escuela D., Angelats E., Etayo Í., Reyes-Resina I., Pulido-Salgado M., Rodríguez-Pérez A.I., Canela E.I., Saura J., Lanciego J.L. (2018). Receptor-heteromer mediated regulation of endocannabinoid signaling in activated microglia. Role of CB1 and CB2 receptors and relevance for Alzheimer’s disease and levodopa-induced dyskinesia. Brain. Behav. Immun..

[269] Labra V.C., Santibáñez C.A., Gajardo-Gómez R., Díaz E.F., Gómez G.I., Orellana J.A. (2018). The neuroglial dialog between cannabinoids and hemichannels. Front. Mol. Neurosci..

[270] Muccioli G.G., Xu C., Odah E., Cudaback E., Cisneros J.A., Lambert D.M., Rodríguez M.L.L., Bajjalieh S., Stella N. (2007). Identification of a novel endocannabinoid-hydrolyzing enzyme expressed by microglial cells. J. Neurosci..

[271] Nomura D.K., Morrison B.E., Blankman J.L., Long J.Z., Kinsey S.G., Marcondes M.C.G., Ward A.M., Hahn Y.K., Lichtman A.H., Conti B. (2011). Endocannabinoid hydrolysis generates brain prostaglandins that promote neuroinflammation. Science.

[272] Barrie N., Manolios N. (2017). The endocannabinoid system in pain and inflammation: Its relevance to rheumatic disease. Eur. J. Rheumatol..

[273] Bruni N., Della Pepa C., Oliaro-Bosso S., Pessione E., Gastaldi D., Dosio F. (2018). Cannabinoid delivery systems for pain and inflammation treatment. Molecules.

[274] Nagarkatti P., Pandey R., Rieder S.A., Hegde V.L., Nagarkatti M. (2009). Cannabinoids as novel anti-inflammatory drugs. Future Med. Chem..

[275] Huggins J.P., Smart T.S., Langman S., Taylor L., Young T. (2012). An efficient randomised, placebo-controlled clinical trial with the irreversible fatty acid amide hydrolase-1 inhibitor PF-04457845, which modulates endocannabinoids but fails to induce effective analgesia in patients with pain due to osteoarthritis of th. Pain.

[276] Wagenlehner F.M.E., van Till J.W.O., Houbiers J.G.A., Martina R.V., Cerneus D.P., Melis J.H.J.M., Majek A., Vjaters E., Urban M., Ramonas H. (2017). Fatty Acid Amide Hydrolase Inhibitor Treatment in Men With Chronic Prostatitis/Chronic Pelvic Pain Syndrome: An Adaptive Double-blind, Randomized Controlled Trial. Urology.

[277] Ostenfeld T., Price J., Albanese M., Bullman J., Guillard F., Meyer I., Leeson R., Costantin C., Ziviani L., Nocini P.F. (2011). A randomized, controlled study to investigate the analgesic efficacy of single doses of the cannabinoid receptor-2 agonist GW842166, ibuprofen or placebo in patients with acute pain following third molar tooth extraction. Clin. J. Pain.

[278] Bradford D., Stirling A., Ernault E., Liosatos M., Tracy K., Moseley J., Blahunka P., Smith M.D. (2017). The MOBILE Study-A Phase IIa enriched enrollment randomized withdrawal trial to assess the analgesic efficacy and safety of ASP8477, a fatty acid amide hydrolase inhibitor, in patients with peripheral neuropathic pain. Pain Med..

[279] Kerbrat A., Ferré J.C., Fillatre P., Ronzière T., Vannier S., Carsin-Nicol B., Lavoué S., Vérin M., Gauvrit J.Y., Le Tulzo Y. (2016). Acute neurologic disorder from an inhibitor of fatty acid amide hydrolase. N. Engl. J. Med..

[280] Moore N. (2016). Lessons from the fatal french study BIA-10-2474. BMJ.

[281] Van Esbroeck A.C.M., Janssen A.P.A., Cognetta A.B., Ogasawara D., Shpak G., Van Der Kroeg M., Kantae V., Baggelaar M.P., De Vrij F.M.S., Deng H. (2017). Activity-based protein profiling reveals off-target proteins of the FAAH inhibitor BIA 10-2474. Science.

[282] Luchicchi A., Lecca S., Carta S., Pillolla G., Muntoni A.L., Yasar S., Goldberg S.R., Pistis M. (2010). Effects of fatty acid amide hydrolase inhibition on neuronal responses to nicotine, cocaine and morphine in the nucleus accumbens shell and ventral tegmental area: Involvement of PPAR-α nuclear receptors. Addict. Biol..

[283] Kawahara H., Drew G.M., Christie M.J., Vaughan C.W. (2011). Inhibition of fatty acid amide hydrolase unmasks CB 1 receptor and TRPV1 channel-mediated modulation of glutamatergic synaptic transmission in midbrain periaqueductal grey. Br. J. Pharmacol..

[284] Maione S., Bisogno T., De Novellis V., Palazzo E., Cristino L., Valenti M., Petrosino S., Guglielmotti V., Rossi F., Di Marzo V. (2006). Elevation of endocannabinoid levels in the ventrolateral periaqueductal grey through inhibition of fatty acid amide hydrolase affects descending nociceptive pathways via both cannabinoid receptor type 1 and transient receptor potential vanilloid type-1 re. J. Pharmacol. Exp. Ther..

[285] Sagar D.R., Kendall D.A., Chapman V. (2008). Inhibition of fatty acid amide hydrolase produces PPAR-α-mediated analgesia in a rat model of inflammatory pain. Br. J. Pharmacol..

[286] Saghatelian A., McKinney M.K., Bandell M., Patapoutian A., Cravatt B.F. (2006). A FAAH-regulated class of N-acyl taurines that activates TRP ion channels. Biochemistry.

[287] Fowler C.J. (2004). Oleamide: A member of the endocannabinoid family?. Br. J. Pharmacol..

[288] Hansen H.S., Rosenkilde M.M., Holst J.J., Schwartz T.W. (2012). GPR119 as a fat sensor. Trends Pharmacol. Sci..

[289] Vandevoorde S., Saha B., Mahadevan A., Razdan R.K., Pertwee R.G., Martin B.R., Fowler C.J. (2005). Influence of the degree of unsaturation of the acyl side chain upon the interaction of analogues of 1-arachidonoylglycerol with monoacylglycerol lipase and fatty acid amide hydrolase. Biochem. Biophys. Res. Commun..

[290] Maldonado R., Baños J.E., Cabañero D. (2016). The endocannabinoid system and neuropathic pain. Pain.

[291] Backonja M.M., Stacey B. (2004). Neuropathic pain symptoms relative to overall pain rating. J. Pain.

[292] Butler S., Jonzon B., Branting-Ekenbäck C., Wadell C., Farahmand B. (2013). Predictors of severe pain in a cohort of 5271 individuals with self-reported neuropathic pain. Pain.

[293] Attal N., Lanteri-Minet M., Laurent B., Fermanian J., Bouhassira D. (2011). The specific disease burden of neuropathic pain: Results of a French nationwide survey. Pain.

[294] Serpell M., Ratcliffe S., Hovorka J., Schofield M., Taylor L., Lauder H., Ehler E. (2014). A double-blind, randomized, placebo-controlled, parallel group study of THC/CBD spray in peripheral neuropathic pain treatment. Eur. J. Pain.

[295] Langford R.M., Mares J., Novotna A., Vachova M., Novakova I., Notcutt W., Ratcliffe S. (2013). A double-blind, randomized, placebo-controlled, parallel-group study of THC/CBD oromucosal spray in combination with the existing treatment regimen, in the relief of central neuropathic pain in patients with multiple sclerosis. J. Neurol..

[296] Lynch M.E., Cesar-Rittenberg P., Hohmann A.G. (2014). A double-blind, placebo-controlled, crossover pilot trial with extension using an oral mucosal cannabinoid extract for treatment of chemotherapy-induced neuropathic pain. J. Pain Symptom Manage..

[297] Englund A., Morrison P.D., Nottage J., Hague D., Kane F., Bonaccorso S., Stone J.M., Reichenberg A., Brenneisen R., Holt D. (2013). Cannabidiol inhibits THC-elicited paranoid symptoms and hippocampal-dependent memory impairment. J. Psychopharmacol..

[298] Gomes F.V., Del Bel E.A., Guimarães F.S. (2013). Cannabidiol attenuates catalepsy induced by distinct pharmacological mechanisms via 5-HT1A receptor activation in mice. Prog. Neuro Psychopharmacol. Biol. Psychiatry.

[299] Hilliard A., Stott C., Wright S., Guy G., Pryce G., Al-Izki S., Bolton C., Giovannoni G. (2012). Evaluation of the Effects of Sativex (THC BDS: CBD BDS) on Inhibition of Spasticity in a Chronic Relapsing Experimental Allergic Autoimmune Encephalomyelitis: A Model of Multiple Sclerosis. ISRN Neurol..

[300] van Amerongen G., Kanhai K., Baakman A.C., Heuberger J., Klaassen E., Beumer T.L., Strijers R.L.M., Killestein J., van Gerven J., Cohen A. (2018). Effects on Spasticity and Neuropathic Pain of an Oral Formulation of Δ9-tetrahydrocannabinol in Patients With Progressive Multiple Sclerosis. Clin. Ther..

[301] Toth C., Mawani S., Brady S., Chan C., Liu C., Mehina E., Garven A., Bestard J., Korngut L. (2012). An enriched-enrolment, randomized withdrawal, flexible-dose, double-blind, placebo-controlled, parallel assignment efficacy study of nabilone as adjuvant in the treatment of diabetic peripheral neuropathic pain. Pain.

[302] Ware M.A., Fitzcharles M.-A., Joseph L., Shir Y. (2010). The Effects of Nabilone on Sleep in Fibromyalgia: Results of a Randomized Controlled Trial. Anesth. Analg..

[303] Pini L.A., Guerzoni S., Cainazzo M.M., Ferrari A., Sarchielli P., Tiraferri I., Ciccarese M., Zappaterra M. (2012). Nabilone for the treatment of medication overuse headache: Results of a preliminary double-blind, active-controlled, randomized trial. J. Headache Pain.

[304] Turcotte D., Doupe M., Torabi M., Gomori A., Ethans K., Esfahani F., Galloway K., Namaka M. (2015). Nabilone as an adjunctive to gabapentin for multiple sclerosis-induced neuropathic pain: A randomized controlled trial. Pain Med..

[305] Narang S., Gibson D., Wasan A.D., Ross E.L., Michna E., Nedeljkovic S.S., Jamison R.N. (2008). Efficacy of Dronabinol as an Adjuvant Treatment for Chronic Pain Patients on Opioid Therapy. J. Pain.

[306] Wilsey B., Marcotte T., Deutsch R., Gouaux B., Sakai S., Donaghe H. (2013). Low-dose vaporized cannabis significantly improves neuropathic pain. J. Pain.

[307] Corey-Bloom J., Wolfson T., Gamst A., Jin S., Marcotte T.D., Bentley H., Gouaux B. (2012). Smoked cannabis for spasticity in multiple sclerosis: A randomized, placebo-controlled trial. CMAJ.

[308] Wallace M.S., Marcotte T.D., Umlauf A., Gouaux B., Atkinson J.H. (2015). Efficacy of Inhaled Cannabis on Painful Diabetic Neuropathy. J. Pain.

[309] Whiting P.F., Wolff R.F., Deshpande S., Di Nisio M., Duffy S., Hernandez A.V., Keurentjes J.C., Lang S., Misso K., Ryder S. (2015). Cannabinoids for medical use: A systematic review and meta-analysis. JAMA J. Am. Med. Assoc..

[310] Martín-Sánchez E., Furukawa T.A., Taylor J., Martin J.L.R. (2009). Systematic review and meta-analysis of cannabis treatment for chronic pain. Pain Med..

